# Lipidomics reveals dramatic lipid compositional changes in the maturing postnatal lung

**DOI:** 10.1038/srep40555

**Published:** 2017-02-01

**Authors:** Sydney E. Dautel, Jennifer E. Kyle, Geremy Clair, Ryan L. Sontag, Karl K. Weitz, Anil K. Shukla, Son N. Nguyen, Young-Mo Kim, Erika M. Zink, Teresa Luders, Charles W. Frevert, Sina A. Gharib, Julia Laskin, James P. Carson, Thomas O. Metz, Richard A. Corley, Charles Ansong

**Affiliations:** 1 Biological Sciences Division, Pacific Northwest National Laboratory, Richland, Washington, USA; 2 Physical Sciences Division, Pacific Northwest National Laboratory, Richland, Washington, USA; 3 Center for Lung Biology, University of Washington, Seattle, Washington, USA.; 4 Texas Advanced Computing Center, University of Texas at Austin, Austin, TX 78758, USA.

## Abstract

Lung immaturity is a major cause of morbidity and mortality in premature infants. Understanding the molecular mechanisms driving normal lung development could provide insights on how to ameliorate disrupted development. While transcriptomic and proteomic analyses of normal lung development have been previously reported, characterization of changes in the lipidome is lacking. Lipids play significant roles in the lung, such as dipalmitoylphosphatidylcholine in pulmonary surfactant; however, many of the roles of specific lipid species in normal lung development, as well as in disease states, are not well defined. In this study, we used liquid chromatography-mass spectrometry (LC-MS/MS) to investigate the murine lipidome during normal postnatal lung development. Lipidomics analysis of lungs from post-natal day 7, day 14 and 6–8 week mice (adult) identified 924 unique lipids across 21 lipid subclasses, with dramatic alterations in the lipidome across developmental stages. Our data confirmed previously recognized aspects of post-natal lung development and revealed several insights, including in sphingolipid-mediated apoptosis, inflammation and energy storage/usage. Complementary proteomics, metabolomics and chemical imaging corroborated these observations. This multi-omic view provides a unique resource and deeper insight into normal pulmonary development.

The majority of perinatal deaths are of infants born prematurely (~10% of all births in the U.S. annually^1^), with the incidence of infant death increasing with decreasing gestational age at delivery^2^. Lung immaturity is a major cause of morbidity and mortality in premature infants with respiratory distress syndrome (RDS) most commonly the cause of death within the first 14 days of life (accounting for 49.5% of deaths) as well as within the first month of life (42.8%)^3^. Bronchopulmonary dysplasia, another pulmonary disorder, represents the leading cause of death after 60 days of life^3^.

Strategies to promote maturation of under-developed lungs in premature infants remain an unsolved clinical challenge. A thorough understanding of the molecular mechanisms driving normal lung development is necessary to identify potential mechanisms for promoting alveologenesis and maturation of the under-developed lungs in premature infants. Towards this goal, comprehensive, untargeted omics studies of normal lung development have been conducted, including several transcriptomics analyses and a single proteomics analysis (see review in ref. 4). While there have been lipidomics studies on lung and lung surfactant in the context of disease and/or injury^5^,^6^, to our knowledge no untargeted omics investigation of lipid profiles during normal lung development has been reported.

Lipids are a large and structurally diverse group of biomolecules and are increasingly appreciated to play crucial roles in maintaining energy balance, guiding intercellular communication, and regulating membrane dynamics. In the lung, lipids are highly significant molecules influencing many processes, and for example, are the primary component of pulmonary surfactant, a lipoprotein complex that functions to reduce the surface tension at the air/liquid interface in alveoli and prevent lung collapse^7^. Deficiency of pulmonary surfactant in the immature lung is the primary cause of RDS. Furthermore, variations in species and distributions of lipids in the lungs are implicated in the outcomes of cystic fibrosis^8^, lung cancer^9^, and asthma^10^. Thus, a thorough characterization of the lung lipidome (the totality of lipids in a biological system) is critical to understand lung function, development and disease states.

In this study, we used liquid chromatography coupled with a tandem mass spectrometer (LC-MS/MS) to profile the temporal ontogeny of lipid changes during post-natal lung development employing a label-free relative quantification approach^5^,^11^,^12^. Specifically we examined whole lung homogenates from mice at post-natal day 7 (PND7), 14 (PND14) and 6–8 weeks after birth (adult), spanning early alveolar to adult developmental stages^13^,^14^. Our LC-MS/MS-based lipidomics analysis resulted in identification of 924 lipid species across 21 lipid subclasses, providing a deep and comprehensive view of the lung lipidome during normal development. Furthermore, we complemented these untargeted lipidomics measurements with additional untargeted proteomics (8289 total proteins, Table S2) and metabolomics (178 total metabolites, Table S3) analyses from the same samples to provide a more comprehensive picture of lipid metabolism during normal lung development. These types of multi-omics analyses have been shown to enhance our understanding of biological systems^12^,^15^. Our results confirmed several previously recognized aspects of post-natal lung development and revealed several new insights into sphingolipid-mediated apoptosis, inflammation and energy storage/usage, providing detailed molecular signatures underlying these processes. Importantly, the lipid-based observations were corroborated by corresponding changes in relevant proteins and metabolites as judged from the complementary proteomics and metabolomics measurements of identical samples. Our multi-omic measurement approach offers unique insights into mammalian respiratory system development.

## Results and Discussion

### Lung lipidome analysis and coverage

We used a lipidomics platform consisting of a commercial UPLC system coupled with a Velos Orbitrap mass spectrometer operating in data-dependent MS/MS mode to profile the murine lung lipidome during post-natal development. Specifically we examined whole lung homogenates from mice at post-natal day 7 (PND7), 14 (PND14), and 6–8 weeks after birth (adult) (n = 3, each). The PND7 and PND14 time-points span peak stages of alveologenesis^14^, during which the alveoli that are critical for the air/gas exchange function of the lung form. Alveologenesis is largely completed by post-natal day 28, therefore, the 6–8 week time point used in our study represents the mature lung. Lipid tandem mass spectra were analyzed using LIQUID, an in-house software for lipid identification and quantification. Confident lipid identifications were made by examining the tandem mass spectra for diagnostic fragment ions along with associated acyl chain fragment information. In addition, the mass measurement error, isotopic profile, extracted ion chromatogram, and retention time of each lipid precursor ion was examined.

**Figure 1:**
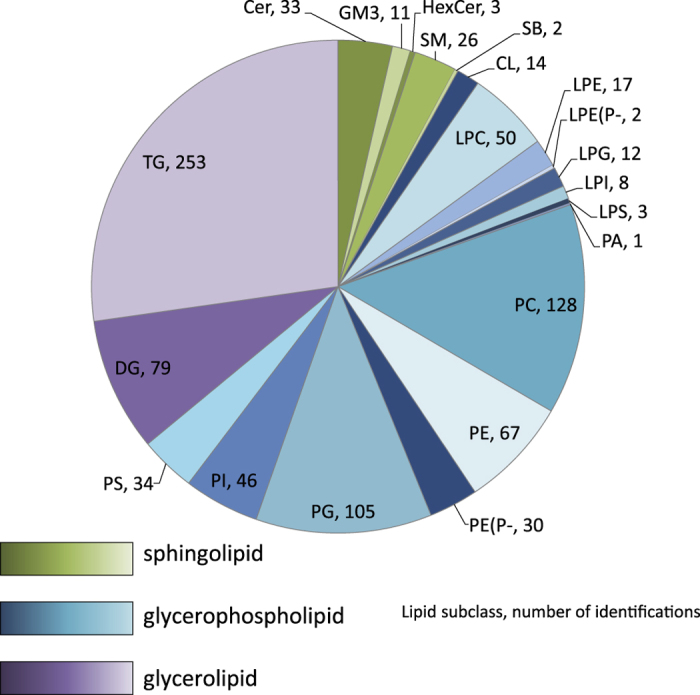
Summary of total lipid identifications color-coded by lipid category and subclass. The majority of lipids identified belonged to the lipid category glycerophospholipid (PL, blue), with the second most belonging to glycerolipid (GL, purple), and the least identifications belonging to sphingolipids (SP, green). The highest number of identifications by lipid subclass belonged to triacylglycerols (TG, 253 identifications), diacylglycerophosphocholines (PC, 128), and diacylglycerophosphoglycerols (PG, 105).

Across all samples, 924 unique lipid species covering 3 lipid categories and 21 lipid subclasses (Fig. 1) were confidently identified based on their MS/MS fragmentation patterns, representing one of the largest lipidome datasets reported to date. The higher lipidome coverage obtained in this study compared to prior studies (see review in ref. 16) likely stems from the use of an UPLC system with a long gradient (90 min) that afforded efficient lipid separation and minimized under-sampling^17^,^18^.

The most commonly identified lipid species in the developing lung lipidome belonged to the triacylglycerol (TG) subclass with 253 identifications, followed by diacylglycerophosphocholine (PC) and the diacylglycerophosphoglycerol (PG) subclasses with 128 and 105 identifications, respectively. Further examination of the data revealed the most abundant species in PC and PG subclasses to be PC(14:0_16:0), PC(16:0_16:0), PG(16:0_18:1), and PG(16:0_16:0), in agreement with a previous report on the lipids known to be most abundant in pulmonary surfactant^19^. Relative quantification of the identified lipids^5^,^11^,^12^ revealed that 161 species varied in a statistically significant manner (t-test; p < 0.05) between the PND7 and PND14 lungs, while 452 lipids and 438 lipids varied in a statistically significant manner (t-test; p < 0.05) between PND7 and adult samples and between PND14 and adult samples, respectively (Table S1). The high degree of dissimilarity between the PND7/PND14 samples versus the adult samples is highlighted by the dendogram in Figure S1, where PND7 and PND14 clustered most closely to each other relative to adult. This trend of a large degree of difference between murine lungs undergoing active alveolarization (PND7/PND14) and murine lungs that had completed alveolarization (adult) was also documented using nanospray desorption electrospray ionization mass spectrometry imaging (nano-DESI MSI) on independent (i.e. obtained from an outside laboratory) murine lung samples (PND7 vs. PND28) (Figure. S2). Focusing on lipids that showed at least a 1.3-fold change between time points from the nano-DESI analysis and also changed in a statistically significant manner between PND7 and adult time points in the LC-MS/MS lipidomics analysis, we defined a subset of 11 lipids for cross comparison between the nano-DESI lipid chemical imaging analysis and the LC-MS/MS lipidomics analysis. Each of the 11 lipids selected had a direction of change across time points as judged from nano-DESI in agreement with observations from the LC-MS/MS lipidomics analysis (Figure S2).

Taken together, our data show broad changes in the murine lung lipidome spanning alveologenesis to adulthood and provide an unprecedented detailed compendium of temporal changes occurring in lipid species during post-natal lung development.

### Characterization of specific lipid categories

#### Sphingolipids

Within the sphingolipid category, 75 unique lipids were identified covering five sphingolipid subclasses. Sphingomyelin (SM) species (26 identifications), underwent the greatest amount of change, with 67% significantly increased (t-test; p < 0.05) in the PND7 sample compared to the adult sample and 25% significantly decreased (t-test; p < 0.05) in the same comparison (Tables S1–9, Figure S3). Of the gangliosides, only GM3 species were detected (11 identifications); all except two decreased significantly (p < 0.05) from the PND7 and PND14 time-points to the adult time-point (Tables S1–10, Figure S4). Sphingosine (d18:1) and sphinganine (d18:0) remained relatively unchanged from PND7 to PND14 and then decreased in the adult time-point (Figure S5). Approximately 75% and 55% of identified ceramides (Cer) changed in a statistically significant manner (t-test; p < 0.05) from PND7 to adult and from PND14 to adult, respectively (37% changed between PND7 and PND14), with a relatively even distribution between lipids increasing or decreasing over this time-frame (Tables S1–11, Figure S6).

#### Glycerolipids

**Figure 2:**
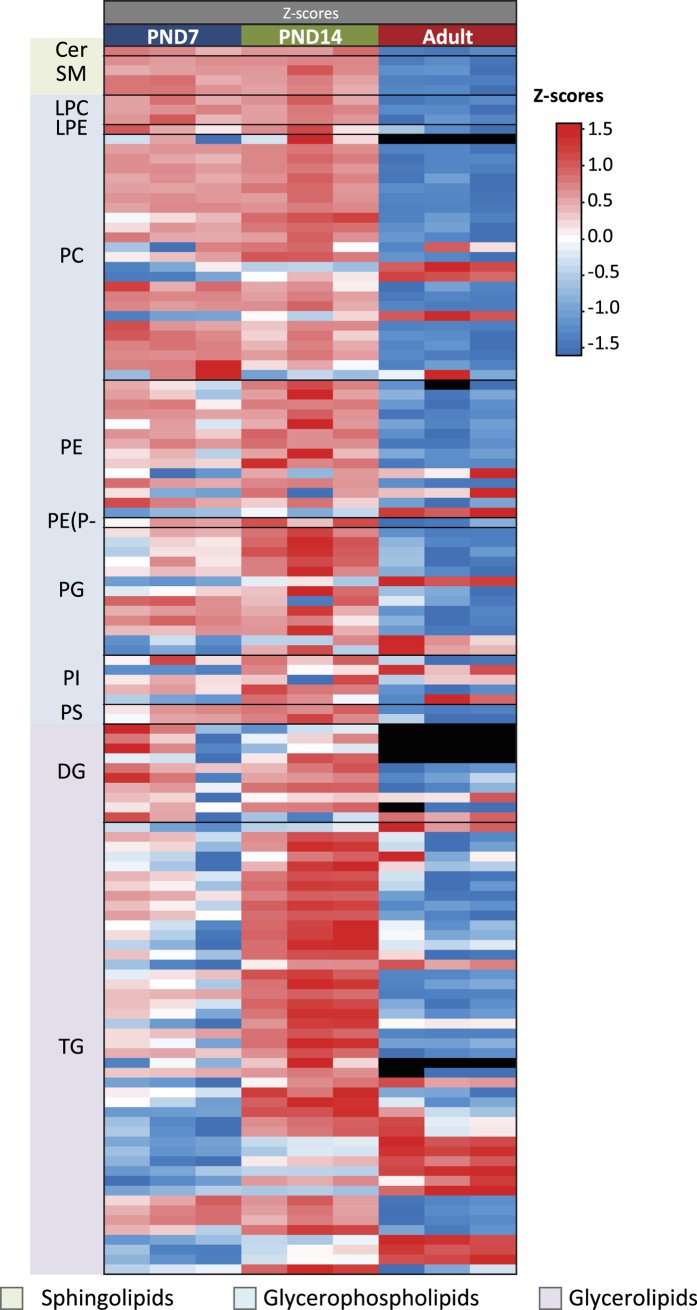
MCSFA are found at highest abundance in PND7 and PND14 murine lungs as compared to adult lungs. All intact lipid species with at least one MCSFA and no coeluting species without MCSFA are displayed. Data in heatmap is z-scored and sorted by component 1 of the principal component analysis. Developmental age is shown on the upper x-axis. Each row represents the normalized intensities of a unique chromatographic feature. The features are color coded by row with red indicating high intensity, blue indicating low intensity, and black indicating below limit of detection (LOD, see color key). The underlying numerical data, including lipid species name, used to generate Figure 2 along with statistical significance values is available in Tables S1–7.

**Figure 3:**
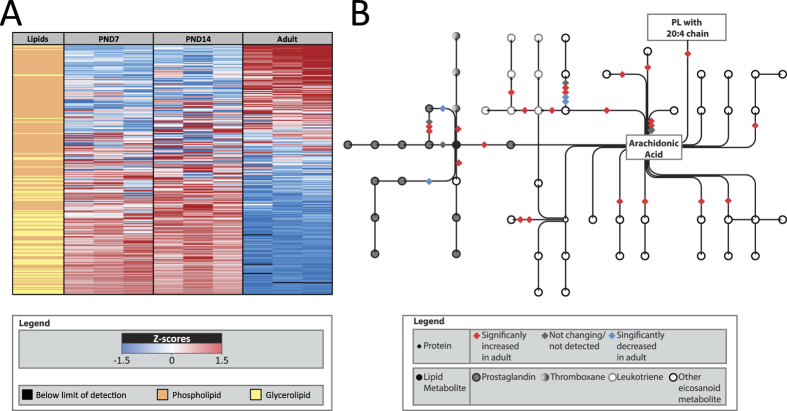
LCPUFA abundance in lipid categories and metabolic pathway facilitating the breakdown into eicosanoid products. (**A**) The majority of intact lipids with LCPUFA are glycerolipids in the young samples in contrast to the high LCPUFA content in phospholipids in the adult samples. Data in heatmap is z-scored and is sorted by component 1 of the principal component analysis. Developmental age is shown on the upper x-axis. Each row represents the normalized intensities of a unique chromatographic feature. The features are color coded by row with red indicating high intensity, blue indicating low intensity, and black indicating below limit of detection (LOD, see color key). Data shown are all identified lipid species with a fatty acid chain with at least 4 double bonds and at least 20 carbons. Lipid species for which the intensity fell between the LOD for all three replicates of one time point were removed. The underlying numerical data, including lipid species name, used to generate Figure 3a along with statistical significance values is available in Tables S1–8. (**B**) Metabolic pathway of arachidonic acid metabolism (mmu:00590). The entire metabolic pathway is shown to be increased in adults as compared to PND7 and PND14 mice. Proteins are indicated by diamonds, lipids and lipid metabolites are indicated by circles. Red diamond indicates highest abundances were observed in adult samples, blue diamond indicates highest abundances were observed in PND7 samples, gray diamond indicates that the species was not changing in a statistically significant manner. The lipid metabolites are color-coded by sub-type of eicosanoid metabolite. The entire arachidonic acid metabolic pathway, starting with cleavage from intact phospholipids, is enriched in adults as compared to the younger samples. Expanded view of pathway with protein names and underlying numerical data used to indicate protein expression patterns in the figure as well as statistical significance values is available in Figure S11 and Table S2.

A total of 332 lipids in triacylglycerol (TG) and diacylglycerol (DG) subclasses were identified. The largest change in glycerolipids occurred from PND14 to adult with 78% of the quantified glycerolipids changing in a statistically significant manner (t-test; p < 0.05) (Table S1). Both TGs and DGs with medium chain saturated fatty acids (MCSFA; 8:0, 10:0, 12:0, 14:0) (Fig. 2, Tables S1–7) and long chain polyunsaturated fatty acids (LCPUFA; greater than 20 carbons and with 4 or more double bonds in at least one fatty acid chain) were more abundant in lungs from PND7 and PND14 mice relative to adult mice (Fig. 3, Tables S1–8). Further, PND7 lungs contained more TGs with longer fatty acid chains, relative to adult lungs – the median total number of carbons in TG acyl chains for PND7 samples is 58 per intact lipid as compared to 52 for adult samples (Tables S1–12, Figure S7). Additionally the TG species identified in PND7 mice had on average almost 3 times as many double bonds as compared to adult mice – the median number of double bonds in TGs from PND7 samples is 8, whereas the median number of double bonds in adult samples is 3 (Tables S1–12, Figure S7).

#### Glycerophospholipids

517 unique glycerophospholipids were identified, spanning 14 subclasses and which exhibited a wide variety of trends supportive of diverse roles for glycerophospholipids during lung development. A major trend was MCSFA being observed in highest abundance in PND7 and PND14 samples relative to adult samples (Tables S1–7, Fig. 2). Additionally, the majority of glycerophosphoethanolamine plasmalogen (PE(P-)) species (17 of 28) increased in abundance over time, whereas only 1 species decreased in abundance in a statistically significant manner (Tables S1–13, Figure S8). Lastly, 46% monoacylglycerophosphocholines (LPCs) statistically increased (t-test; p < 0.05) from the PND7 to the adult time-point, whereas only 20% of LPCs decreased over the same time span (Tables S1–14, Figure S9). The LPC species that were most abundant in PND7 and PND14 samples tended to have either a MCSFA or a LCPUFA.

### Sphingolipid-mediated apoptosis revealed at early alveolar stage of lung development

Sphingolipids are a class of lipids that are intricately involved in numerous cell processes, including proliferation, senescence, differentiation, and signaling^20^. In the lung, sphingolipids have been used both as a marker for pulmonary development as well as for disease^21^. Ceramides, sphingosine, and sphingosine-1-phosphate (S1P) determine an apoptotic balance within cells, in which relative increases in ceramides and sphingosine result in cell death^22^,^23^. Further, ceramides with different chain lengths have different effects on the cell; some species promote proliferation, some inhibit proliferation, and others can induce autophagy or apoptosis^20^.

**Figure 4:**
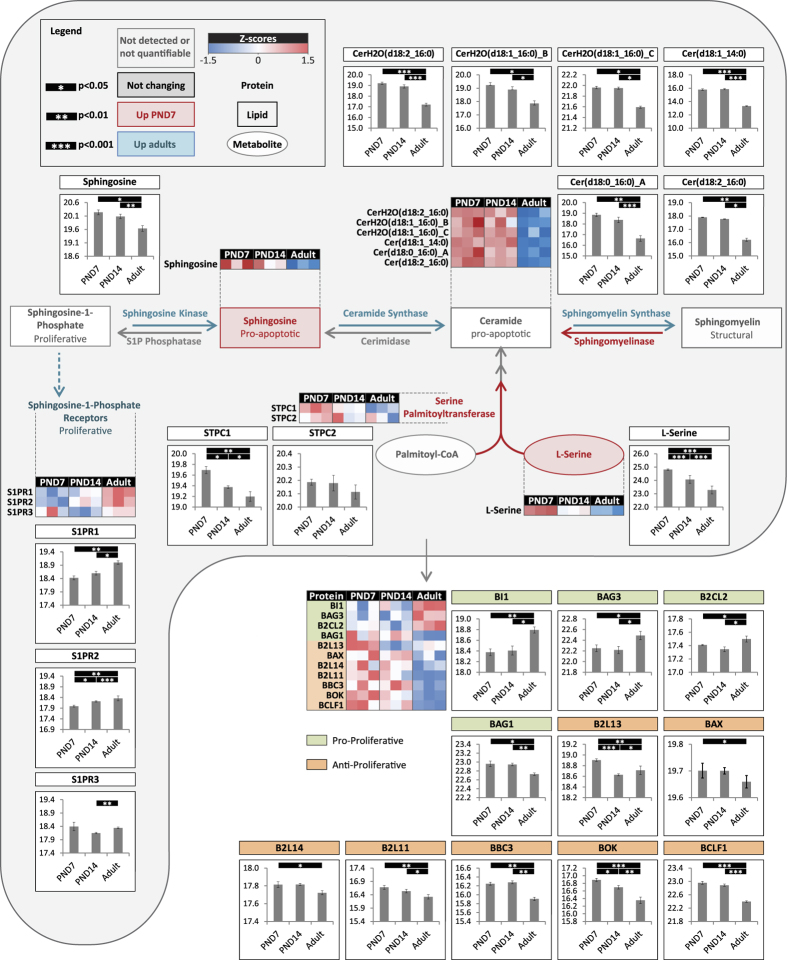
Pro-apoptotic sphingolipid species are observed at highest abundance in mouse lungs undergoing active alveolarization as compared to adult lungs. L-serine (highest PND7) and palmitoyl-CoA are combined by serine palmitoyltransferase (highest PND7) to begin the *de novo* pathway of ceramide synthesis. Once ceramide is formed, ceramide species with chain lengths of 14:0, 16:0 and 18:0 are known to induce apoptosis and autophagy (shown in the ceramide heatmap: all pro-apoptotic/autophagic ceramides that change in a statistically significant manner between PND14 and adult). Sphingosine is also a pro-apoptotic molecule that is detected at highest abundance in PND7 mice. This is in contrast to the protein receptors for proliferative sphingosine-1-phosphate (all receptors detected shown in heatmap, S1PR 1–3), which are highest in the adult mice. Sphingolipid-mediated apoptosis is then known to involve the BCL-2/BAX proteins. Shown in the heatmap are the pro-proliferative vs. anti-proliferative BCL-2/BAX family that change in a significantly significant manner between PND14 and adult. All data taken together suggest that sphingolipid-mediated apoptosis occurs in lung tissue undergoing alveolarization. Bar graphs are provided for each lipid, metabolite and protein present in the figure with significance of differential expression across time indicated by stars (see figure legend). The y-axis of each bar graph represent normalized log2 intensity values. The underlying numerical data used to generate Figure 4 along with statistical significance values is available in Tables S1–3 for lipids, Table S2 for proteins and Table S3 for metabolites. In the figure legend, z-score colour coding refers to heatmaps, whereas colour coding for ‘Up PND7’ and ‘Up adults’ refers to arrows and shapes indicating lipids and metabolites.

In this study, we observed an increase in pro-apoptotic and pro-autophagic ceramide species (Cer 14:0, Cer 16:0, and Cer 18:0) and pro-apoptotic sphingosine in lungs of PND7 and PND14 mice^24^,^25^ (Fig. 4, Tables S1–3). Although S1P was not detected in our study, a corresponding proteomics analysis of the same samples (see Supplementary Methods) revealed the sphingosine-1-phosphate receptors (S1PR1, S1PR2 and S1PR3 detected), which mediate the proliferative/protective effects of S1P^26^, all exhibited lower abundance in lungs from PND7 and PND14 mice relative to those from adult mice (Fig. 4, Table S2). In the lung specifically, S1P and the S1PRs are known to have strong effects on maintenance of the endothelial barrier^27^, with the ability to rearrange the cytoskeleton to alter vascular permeability^28^. Corresponding metabolomics analysis of the same samples (see Supplementary Methods) showed the ceramide precursor L-serine increased in PND7 relative to adult lungs (Fig. 4, Table S3), with the corresponding proteomics analysis (described above) showing serine palmitoyltransferase, the rate-limiting enzyme in *de-novo* ceramide synthesis from serine, increased in PND7 relative to adult samples (Fig. 4, Table S2). Taken together, the above multi-omic observations support the notion of a shift in balance towards a more apoptotic state in the PND7 samples (Fig. 4). Additionally, almost all GM3 lipids were detected in higher abundance in PND7 and PND14 samples, relative to adult. In some tissues, these lipids are known to have pro-apoptotic effects; however, more research is necessary to elucidate the precise role of GM3 lipids in the lung^29^,^30^. Recent studies have shown that BCL-2 genes are downregulated relative to BAX genes in association with sphingolipid-mediated cell death, which promotes apoptosis^31^,^32^. The corresponding proteomics data in this study revealed that more pro-apoptotic BCL-2 and BAX family proteins were elevated in PND7 and PND14, mice while more proliferative proteins were elevated in the adult mice (Fig. 4, Table S2).

The increase in pro-apoptotic molecules in samples from PND7 and PND14 mice is likely related to tissue remodeling and thinning associated with alveolarization and gas exchange optimization^33^. Previous studies have shown that during alveolarization as many as 20% of fibroblasts eventually undergo apoptosis in rat lungs^32^, and up to 12% of mesenchymal and epithelial cells in rats at postnatal day 1 are undergoing apoptosis^34^. Our multi-omics data support these earlier observations of increased apoptosis during alveolarization and for the first time provide a detailed view of the molecular signatures driving this process.

### Differential modulation of inflammation mediating molecules during lung development

Complex lipids containing LCPUFA, in particular glycerophospholipids within cell membranes, have been linked to inflammatory processes^35^,^36^. These LCPUFA can impact the inflammatory process multiple ways including influencing membrane fluidity, protein function, and also acting as substrates for the generation of lipid signaling molecules (i.e. lipid mediators); the latter of which have potent effects on the regulation of inflammation in tissues and are themselves tightly regulated within a system^37^. These bioactive lipid mediators are generated by the actions of phospholipases on intact lipids containing 20:4, 20:5, and 22:6 fatty acids, which then release arachidonic acid (AA), eicosapentaenoic acid (EPA), or docosahexaenoic acid (DHA), respectively^38^. These fatty acids are then enzymatically modified by cytochrome P450 proteins, cyclooxygenases, and lipoxygenases, among others, to create pro-inflammatory, anti-inflammatory, and pro-resolution bioactive lipid mediators^37^. Although many bioactive lipid mediators can have both pro-inflammatory and pro-resolution effects (i.e. lipoxins), it is generally accepted that derivatives of arachidonic acid (AA, 20:4) tend to be pro-inflammatory, whereas derivatives of eicosapentaenoic acid (EPA, 20:5) and particularly docosahexaenoic acid (DHA, 22:6) tend to be pro-resolution^39^.

We found increased glycerophospholipids with LCPUFA in adult mice as compared to PND7 and PND14 mice, with the PND7 and PND14 mice having most LCPUFAs localized in glycerolipid species (Fig. 3A, Tables S1–8). The corresponding proteomics data revealed lipases (diacylglycerol lipase alpha, phospholipase A2, adipose triglyceride lipase) that cleave LCPUFA from the intact lipids were increased in adult mice as compared to the PND7 and PND14 mice. Additionally, the proteins (cytochrome P450, cyclooxygenases, and lipoxygenases) that then generate the bioactive lipid mediators from the cleaved fatty acids were also found in higher abundance in the lungs from adult mice (Table S2). In the arachidonic acid (AA) metabolic pathway alone, 70% of proteins detected were significantly more abundant (p < 0.05) in adult (Fig. 3B, Figure S11). Taken together with the increased glycerophospholipids with LCPUFA in adult mice, this suggests that the source of arachidonic acids likely derives from glycerophospholipids in this study. Indeed, it is well known that PCs and PIs act as sources for arachidonic acids. For example, the protein PA24A selectively hydrolyzes arachidonyl phospholipids in the sn-2 position releasing arachidonic acid^40^. These data collectively highlight the ability of multi-omics analyses to provide insight on the complex interplay of molecules that regulate inflammation in the lungs during development.

In addition to LCPUFA, lysoPCs (LPC) were more abundant in adult animals (Figure S9, Tables S1–14). Several studies have shown that LPC species are involved in pro-inflammatory processes in situations of acute injury as well as chronic stress^41^. LPCs are produced by the actions of phospholipase A2 (PLA2) and aid in the recruitment of T-lymphocytes^42^, promote production of pro-inflammatory cytokines by immune cells^43^,^44^, increase reactive oxygen species production^45^, and upregulate cell adhesion molecules^46^. In the lungs, LPCs increase the permeability of the alveolar epithelium^47^ and inactivate surfactant function^48^. In our study, the majority of LPC species were more abundant in lungs from adult mice (Figure S9, Tables S1–14), correlating with the increase in phospholipase A2 (PLA2) in the adult lungs.

Plasmalogen species are lipids that modulate membrane structure^49^. In addition, due to the instability of the vinyl ether bond connecting the glycerol backbone to the fatty acid in plasmalogen species, these lipids are able to act as lipophilic antioxidants in cells^50^,^51^. Plasmalogens can have a protective effect against iron-induced lipid peroxidation, and it has been suggested that they are some of the most significant antioxidants in the lung and pulmonary surfactant^50^,^51^. In our study, glycerophosphoethanolamine plasmalogen (PE(P-)) species were found in highest abundance in lungs from adult mice (Figure S8, Tables S1–13). Together with the above observations related to LCPUFAs and LPCs, the increased (PE(P-)) levels in adult animals supports the notion that the adult lungs are in a greater state of inflammation, with a carefully coordinated interaction between pro-inflammatory and pro-resolution lipids and associated protein mediators to maintain a finely balanced homeostasis to prevent overt injury to the lung.

### A role for medium chain saturated fatty acids (MCSFA) as a rapid source of energy in the early alveolar stage

For each lipid subclass in which MCSFA were detected (Cer, DG, LPC, LPE, PC, PE, PE(P-, PG, PI, PS, SM, TG), the highest abundances were observed in lungs from PND7 and PND14 mice, relative to those from adults. Specifically, of the 92 lipid species that were detected with at least one MCSFA and no co-eluting species lacking MCSFA, 88% were more abundant in young relative to adult mice (Fig. 2, Tables S1–7). The higher abundance of MCSFA in younger mice may suggest a role for MCSFA as a rapid energy source in the earlier alveolar stage. Free MCFAs do not need to be transported by carnitine palmitoyl transferase to the mitochondria in order to undergo beta-oxidation; because of this they are preferentially oxidized over long chain fatty acids for energy^52^. Oxidation of free MCFAs may also contribute to a ketotic state, because an increase in beta-oxidation of fatty acids results in an increase in ketone bodies^53^,^54^. In our study, an increase in beta-hydroxybutyrate, a ketone body, was observed in metabolomics data from PND7/PND14 lung tissue as compared to adult (Table S3 and Figure S10), suggesting a high rate of fatty acid oxidation in the younger animals (acetone and acetoacetate, the other two ketone bodies, were not detected; Table S3)). Recent work has shown that mild ketosis is frequently observed in suckling mammalian infants and that ketone bodies are vital to organ development, particularly the brain^55^. Ketone bodies are preferentially utilized over glucose for *de novo* surfactant production in lungs of newborn rats^56^. These observations suggest that MCSFAs may be important for newborn lung development, primarily by providing a source of rapidly available energy or intermediates for new and evolving postnatal functions.

## Conclusion

In this study, we used an untargeted lipidomics approach to provide a comprehensive picture of the murine lipidome throughout alveolarization, resulting in the confident identification (i.e. based primarily on MS/MS fragmentation patterns) of over 900 lipids in 21 lipid subclasses and representing one of the largest lipidome datasets reported to date. The data showed large global shifts in the lipidome over time and provided insight into the molecular level events driving cellular processes and function. Specifically, differential lipid abundances were observed in molecular species that are known to have significant roles in regulating apoptosis, inflammation, and energy storage. These observations were supported by data from complementary proteomics and metabolomics analysis on the same samples, as well as lipid chemical imaging analysis. Our work highlights the utility of leveraging deep lipidome measurements and complementary multi-omic data to provide unique insights into pulmonary development and potentially discover therapeutic targets to promote lung maturation.

## Materials and Methods

### Animal care for multi-omic analyses

All animal care procedures were approved by PNNL Institutional Animal Care and Use Committee, and carried out in accordance with PNNL Institutional Animal Care and Use Committee guidelines and regulations. A total of 6, 6–7 week old mice and 6 time-mated C57BL/6 J mice were ordered from The Jackson Laboratory and kept in standard shoebox caging (72 °F ± 3°, humidity 50% rH) with ALPHA-dri^®^ bedding (Shepherd Specialty Papers) at Pacific Northwest National Laboratory. The light-dark cycle was 12 hours each and the mice were fed ad libitum food (Lab Diet 5002 Certified Rodent Diet) and water. Mice were euthanized by cervical dislocation (>PND14) or decapitation (PND7) and exsanguinated via an incision in the left ventricle.

### Dissection and homogenization

The heart and lungs were washed *in situ* with an injection of 10 ml of 4 °C PBS (with Ca2+ and Mg2+) through the right ventricle. The lungs were then removed en bloc and washed twice in cold PBS. Any contaminating tissue was carefully removed at this point before flash freezing in liquid nitrogen and storage at −70 °C until further sample prep. Frozen tissue samples were transferred into a tarred, pre-chilled Eppendorf Safe-Lock tube and the total mass was determined. The tissue samples were then homogenized using a Qiagen TissueLyser II with a 2 × 24 adapter (chilled to −20 °C) following the vendor suggested protocol for tissue samples, with modifications. In short, a 3 mm tungsten bead was added to each tube along with 0.5 mL of chilled methanol prior to processing twice in TissueLyser II for 3 minutes at 30 Hz. After homogenization, the samples were again chilled to −20 °C prior to transferring to a chilled Sorenson MμlTI™ SafeSeal™ microcentrifuge.

### Modified Folch extraction

The aqueous polar metabolites, lipids, and proteins were extracted from each homogenate using a modified Folch extraction^57^ as previously described^58^. Keeping each sample on ice, a volume of chilled chloroform and water were added to a final ratio of 3:8:4 water-chloroform-methanol, mixing gently after each addition. The samples were chilled on ice for 5 minutes before mixing well and separating the layers by centrifugation (10 k × g, 10 minutes, 4 °C). The aqueous polar metabolites, lipids and proteins were isolated and concentrated to dryness in a vacuum concentrator and stored at −70 °C until ready for further processing.

### Lipid sample preparation and analysis by LC-MS/MS

The lower organic layer of the Folch extraction was reconstituted in MeOH and subjected to LC-ESI-MS/MS analyses, using a Waters NanoAquity UPLC system (Waters column, HSS T3 1.0 mm × 150 mm × 1.8 μm particle size) interfaced with a Velos-ETD Orbitrap mass spectrometer (Thermo Scientific, San Jose, CA). Data dependent scan events occurred both in the ion trap (collision-induced dissociation, CID) and Orbitrap (HCD) using normalized collision energy (NCE) of 30 and 35 arbitrary units, respectively, in the same run as outlined previously^59^. Scan events were between the mass ranges of m/z 200–2000 for both positive and negative ionization. A full scan event occurring in the Orbitrap with a resolving power of 60,000. The full scan event is followed by MS/MS of the top 6 ions, alternating between HCD (resolving power of 7500) and CID, respectively, with an isolation width of 2 and dynamic exclusion of 60 sec. The ion time for the full scan MSn was 500 ms, for MS/MS in the Orbitrap (HCD) 1000 ms, and MS/MS in the ion trap (CID) 50 ms. The automatic gain control for the full scan was 1,000,000, for HCD MS/MS 50,000, and for CID MS/MS 10,000. Unique lipid species were separated chromatographically over a 90 min gradient elution (mobile phase A: ACN/H2O (40:60) containing 10 mM ammonium acetate; mobile phase B: ACN/IPA (10:90) containing 10 mM ammonium acetate) at a flow rate of 30 μl/min. The LC gradient details can be found in the supplementary Tables S1–6. Higher-energy collision dissociation (HCD) and collision-induced dissociation (CID) were used to analyze samples in both positive and negative ionization modes in order to gain high coverage of lipid species.

### Lipid data processing

LC-MS/MS raw data files were imported into the in-house developed software LIQUID (Lipid Informed Quantitation and Identification) for semi-automated identification of lipid molecular species. Lipid identifications were confirmed by examination of the isotopic profiles, precursor masses, XICs, and tandem mass spectra. In the tandem mass spectra, the diagnostic ion in addition to fatty acid fragment ions were used to confirm the identification. Each ionization mode of the datasets was then separately aligned and gap-filled based on m/z and retention time using MZmine 2^60^, with manual verification of every feature. Peak apex intensities were exported for statistical analysis. All lipid nomenclature follows the “Comprehensive Classification System for Lipids” developed by the International Lipid Classification and Nomenclature Committee^61^,^62^.

### Protein sample preparation and analysis by LC-MS/MS

Extracted proteins were denatured, alkylated, digested with trypsin and desalted on a C18 SPE cartridge (Discovery C18, 1 mL, 50 mg, Sulpelco). The peptide concentration was measured by BCA assay (Thermo Scientific). Peptides were labeled with 10-plex TMT reagents (Life technology) according to the manufacturer’s instructions prior to be pooled together. They were then separated using an off-line reversed-phase chromatography column as previously described^63^. 24 fractions were collected. 5 μL of 0.1 μg/μL of peptides from each fraction were analyzed by reverse phase LC-MS/MS using a Waters nanoEquityTM UPLC system (Millford) coupled with a Q-Exactive mass spectrometer (Thermo Scientific).

### Proteomics LC-MS/MS system

The LC was configured to load the sample first on a solid phase extraction (SPE) column followed by separation on an analytical column. Analytical columns were made in-house by slurry packing 3-μm Jupiter C18 stationary phase (Phenomenex, Torrence, CA) into a 70-cm long, 360 μm OD × 75 μm ID fused silica capillary tubing (Polymicro Technologies Inc., Phoenix, AZ). Samples were loaded on the SPE column via a 5 μL sample loop for 30 minutes at a flow rate of 3 μL per minute and then separated by the analytical column using a 110 minute gradient from 99% mobile phase A (MP-A) to 5% MP-A at a flow rate of 0.3 μL per minute. Mass spectrometry analysis was started 15 minutes after the sample was moved to the analytical column. After the gradient was completed, column was washed with 100% mobilie phase B (MP- B) first and then reconditioned with 99% MP- A for 30 minutes. The effluents from the LC column were ionized by electrospray ionization and mass analyzed with a QExactive hybrid quadrupole/Orbitrap mass spectrometer operated in the data-dependent analysis mode. Top 10 ions from the survey scan were selected by a quadrupole mass filter for high-energy collision dissociation (HCD) in collision with nitrogen and mass analyzed by the Orbitrap. An isolation window of 2 Daltons was used for the isolation of ions and a collision energy of 28% was used for HCD with AGC setting of 1e5 ions. Mass spectra were recorded for 100 minutes by repeating this process with a dynamic exclusion of previously selected ions for 60 seconds.

### Proteomics data analysis

Raw mass spectrometry data were converted to peak lists (DTA files) using the DeconMSn (version 2.3.1.2) and searched with MS-GF+^64^ against Uniprot/SwissProt mus musculus database (downloaded 2013-09-18), bovine trypsin and human keratin sequences. The identified spectra were filtered based on their MSGF+ scores and only the proteins with two proteospecific peptides were conserved resulting in a protein and peptide false discovery rate <1%. For the quantitative analysis, the TMT reporter ion intensities were extracted with MASIC^65^. TMT reporter intensities were summed from the different peptides belonging to the same proteins. Proteins with missing data were excluded for the quantification analysis.

### Polar metabolite sample preparation and analysis by GC-MS

Analysis of the dried polar metabolites was performed as previously described^66^. Prior to analysis, the carbonyl groups were protected by treating with 20 uL of methoxyamine solution (30 mg/mL in pyridine) and incubated for 90 minutes at 37 °C with shaking. The hydroxyl and amine groups were then derivatized with 80 uL of N-methyl-N-(trimethylsilyl) trifluoroacetamide (MSTFA) with 1% trimethylchlorosilane (TMCS), incubating for 30 minutes at 37 °C with shaking. The samples were then allowed to cool to room temperature and were analyzed by an Agilent GC 7890 A coupled with MSD 5975 C mass spectrometer (Agilent Technologies, Santa Clara, CA). Separations were performed on a HP-5MS column (30 m × 0.25 mm × 0.25 μm; Agilent Technologies). The injection mode was splitless, and the injection port temperature was held at 250 °C. The column oven was initially maintained at 60 °C for 1 min and then ramped to 325 °C by 10 °C/min, followed by a 10 min hold at 325 °C.

### Polar metabolite data analysis

GC-MS raw data files from each Experiment were processed using the Metabolite Detector software, version 2.5 beta. Briefly, Agilent. D files were converted to netCDF format using Agilent Chemstation, followed by conversion to binary files using Metabolite Detector (PMID: 19358599). Retention indices (RI) of detected metabolites were calculated based on the analysis of the FAMEs mixture, followed by their chromatographic alignment across all analyses after deconvolution. Metabolites were initially identified by matching experimental spectra to an PNNL augmented version of FiehnLib^67^, containing spectra and validated retention indices for over 850 metabolites, using a Metabolite Detector match probability threshold of 0.6 (combined retention index and spectral probability). All metabolite identifications were manually validated to reduce deconvolution errors during automated data-processing and to eliminate false identifications.

### Normalization and statistics

For lipidomics, metabolomics and proteomics, the data was log transformed and median normalized within each sample and statistically significant changes were determined using two-tailed, homoscedastic t-test and/or ANOVA using R stats package. Pearson’s correlation and hierarchical clustering were also performed using this stats package. The principal component analysis were realized using the ‘mixOmics’ package^68^.

### Nano-DESI-Mass Spectrometry Imaging Tissue Collection, and Handling

All mice used for imaging data were housed in the Cincinnati Children’s Hospital Medical Center Animal Care Facility according to National Institutes of Health and institutional guidelines for the use of laboratory animals. All protocols of the present study were reviewed and approved by Cincinnati Children’s Hospital Research Foundation Institutional Animal Care and Use Committee. C57BL/6 mice from JAX Mice (Jackson Laboratory, Bar Harbor, ME, USA) were sacrificed at day 7 (PND7 - early alveolar) and day 28 (PND28 - late alveolar) by CO2 overdose. Lungs were collected, cleaned, embedded in carboxymethyl cellulose (CMC) and stored in −80 °C freezer. Samples were sectioned into 10 μm thick slices with a Thermo CryoStar NX70 (Thermo Scientific, Waltham, MA) microtome to generate coronal sections of the lungs. Sections were thaw-mounted onto regular glass slides and stored at −80 °C until analysis. The section was allowed to thaw at room temperature right before mounting onto the nano-DESI sample holder for analysis.

### Nano-DESI-Mass Spectrometry Imaging analysis

A custom made nano-DESI source^69^ comprised of a sample holder attached to a high-resolution motorized XYZ sample stage (Zaber Technologies, Vancouver, BC) controlled via a custom-designed Labview software^69^ was mounted onto an XL LTQ/Orbitrap mass spectrometer (Thermo Scientific, Waltham, MA). The OD 150 μm × ID 50 μm fused silica capillaries were used to make both nano-DESI primary and secondary capillaries. A solvent consisting of 90% methanol (Fisher Scientific) and 10% water (HPLC grade, Fisher Scientific) was used with the addition of 3 μM of LPC 19:0 and PC 23:0 as internal standards. Solvent was delivered at a flow rate of 500 nl/min with an applied voltage of 3.5 kV through the primary capillary. The heated capillary inlet was held at 30 V and 250 °C. The primary and secondary capillaries were positioned using micromanipulators (XYZ 500MIM, Quater Research and Development, Bend, OR) under monitoring of two Dino-Lite digital microscopes (AnMo Electronics Corporation, Sanchong, New Taipei, Taiwan).

### Nano-DESI-Mass Spectrometry Imaging data collection and image processing

For each time point, three biological and three technical replicates were acquired in both positive and negative mode. Imaging experiments were performed by scanning the sample line by line under the nano-DESI probe at a constant velocity while acquiring mass spectra. The imaging scan rate was 70 μm/s and spacing between lines was set at 100 μm. The nano-DESI stage and mass spectrometer was synchronized by triggering at the beginning of each line scan. The mass spectrometer was operated with a mass resolution of 60 000 (m/Δm) at m/z 400. Mass spectral data acquired by the Xcalibur software were subsequently processed by MSI QuickView^70^, a visualization software developed at PNNL.

## Additional Information

**Accession codes**: Data deposited and freely available at ProteomeXchange data repository, ProteomeXchangeID: PXD004651 and MassIVE data repository, MassIVE ID: MSV000080000.

**How to cite this article**: Dautel, S. E. *et al*. Lipidomics reveals dramatic lipid compositional changes in the maturing postnatal lung. *Sci. Rep.* **7**, 40555; doi: 10.1038/srep40555 (2017).

**Publisher's note:** Springer Nature remains neutral with regard to jurisdictional claims in published maps and institutional affiliations.

## Supplementary Material

Supplementary Information

Supplementary Dataset 1

Supplementary Dataset 2

Supplementary Dataset 3
