# Supplementary material for: Lipidomics reveals dramatic lipid compositional changes in the maturing postnatal lung
Source: Sci Rep. 2017 Feb 1;7:40555. doi: 10.1038/srep40555 (PMC5286405; doi:10.1038/srep40555)
Supplement: Supplementary Information [file srep40555-s1.pdf]

Lipidomics reveals dramatic lipid compositional changes in the maturing postnatal lung

Sydney E. Dautel[a#], Jennifer E. Kyle[a#], Geremy C. Clair[a], Ryan L. Sontag[a], Karl K. Weitz[a], Anil K. Shukla[a], Son N. Nguyen[b], Young-Mo Kim[a], Erika M. Zink[a], Teresa Luders[a], Charles Frevert[c], Sina A. Gharib[c], Julia Laskin[b], Thomas O. Metz[a], Richard A. Corley[a] and Charles Ansong[a*].

a. Biological Sciences Division and b. Physical Sciences Division, Pacific Northwest National Laboratory, Richland, Washington c. Center for Lung Biology, University of Washington, Seattle, Washington

* Correspondence to charles.ansong@pnnl.gov

# Contributed equally and considered co-first authors

Supplementary Figure Legends

Figure S1. Hierarchical clustering of data collected in positive and negative ionization modes of PND7, PND14 and adult mice. R stands for replicate number. Data show high degree of similarity between PND7 and PND14 time points, as compared to adults.

Figure S2. Subset of lipids identified in nano-DESI chemical imaging demonstrating the trends of intact lipids with medium chain saturated fatty acids (MCSFA) and long chain polyunsaturated fatty acids (LCPUFA) being more abundant in PND7 and PND14 mice whereas monoacylglycerophosphocholines (LPC) species are more abundant in the adult mice. The lipid with total carbon and double bond count for each image is labeled above the image in black, with all lipids that match the m/z from the LC-MS dataset in gray brackets following.

Figure S3. All identified sphingomyelin (SM) species shown in heatmap format. Data in heatmap is z-scored and sorted by component 1 of the principal component analysis. Developmental age is shown on the upper x-axis. Each row represents the normalized intensities of a unique chromatographic feature. The features are color coded by row with red indicating high intensity, blue indicating low intensity, and black indicating below limit of detection (LOD, see color key). Lines with more than one lipid identification indicate that the listed lipid names were co-eluting. If more than one chromatographic peak was identified with the correct mass, fragmentation pattern, and isotopic profile then the two features (likely isomers) were annotated “lipid_A” and “lipid_B,” according to retention time. The underlying numerical data

used to generate Figure S3 along with statistical significance values is available in Tables S1 – Tab9.

Figure S4. All identified ganglioside (GM3) species shown in heatmap format. Data in heatmap is z-scored and sorted by component 1 of the principal component analysis. Developmental age is shown on the upper x-axis. Each row represents the normalized intensities of a unique chromatographic feature. The features are color coded by row with red indicating high intensity, blue indicating low intensity, and black indicating below limit of detection (LOD, see color key). If more than one chromatographic peak was identified with the correct mass, fragmentation pattern, and isotopic profile then the two features (likely isomers) were annotated “lipid_A” and “lipid_B,” according to retention time. The underlying numerical data used to generate Figure S4 along with statistical significance values is available in Tables S1 – Tab10.

Figure S5. Sphingosine (C18) and sphinganine (C18) are more abundant in PND7 and PND14 murine lungs than adult lungs. Data shown are $\log_2$ normalized abundances of sphingosine (C18) and sphinganine (C18). Error bars show standard error of three replicates. Asterisks denote statistical significance as shown in key.

Figure S6. All identified ceramide (Cer) species shown in heatmap format. Data in heatmap is z-scored and sorted by component 1 of the principal component analysis. Developmental age is shown on the upper x-axis. Each row represents the normalized intensities of a unique

chromatographic feature. The features are color coded by row with red indicating high intensity, blue indicating low intensity, and black indicating below limit of detection (LOD, see color key). Lines with more than one lipid identification indicate that the listed lipid names were co-eluting. If more than one chromatographic peak was identified with the correct mass, fragmentation pattern, and isotopic profile then the two features (likely isomers) were annotated “lipid_A” and “lipid_B,” according to retention time. The underlying numerical data used to generate Figure S6 along with statistical significance values is available in Tables S1 – Tab11.

Figure S7. Triacylglycerides that are statistically more abundant in the PND7 and PND14 murine lungs had a greater number of double bonds and longer fatty acid chains than those that were most abundant in the adult murine lungs. Data shown are all lipids that were identified as being statistically different (p value < 0.05) between PND7 and adult mice. Developmental age is shown on upper x-axis. Each row represents a unique normalized chromatographic feature. The lipid abundance is color coded with blue indicating low intensity and red indicating high intensity (see color key). Data were sorted based on fold change. Lines with more than one lipid identification indicate that the listed lipid names were co-eluting. Lipids were deleted from the table if the compound was below the limit of detection for any of the replicates. Compounds with more total carbons in the fatty acids are darker green, whereas those with less are lighter. Compounds with more double bonds in the fatty acids are darker purple, whereas those with less are lighter (see color key). The underlying numerical data used to generate Figure S7 along with statistical significance values is available in Tables S1 – Tab12.

Figure S8. The abundances of all identified PE plasmalogen species shown in heatmap form. Data in heatmap is z-scored and sorted by component 1 of the principal component analysis. Developmental age is shown on the upper x-axis. Each row represents the normalized intensities of a unique chromatographic feature. The features are color coded by row with red indicating high intensity, blue indicating low intensity, and black indicating below limit of detection (LOD, see color key). Lines with more than one lipid identification indicate that the listed lipid names were co-eluting. If more than one chromatographic peak was identified with the correct mass, fragmentation pattern, and isotopic profile then the two features (likely isomers) were annotated “lipid_A” and “lipid_B,” according to retention time. The majority of PE(P- species are more abundant in the adult samples than in the samples undergoing alveolarization. The underlying numerical data used to generate Figure S8 along with statistical significance values is available in Tables S1 – Tab13.

Figure S9. The abundances of all identified lysoPC species shown in heatmap form with the same specifications as described in Figure 2. Frequently with lyso species, two peaks are observed chromatographically, commonly thought to be SN-1 and SN-2 isomers of the lyso. If two peaks were able to be identified in this dataset, the peak eluting first was annotated “lipid_A” and the second peak was annotated “lipid_B” as shown above. The majority of LPC species are more abundant in the adult mice than in the mice undergoing alveolarization. The underlying numerical data used to generate Figure S9 along with statistical significance values is available in Tables S1 – Tab14.

Figure S10. Beta-hydroxybutyrate is more abundant in PND7 and PND14 murine lungs than adult lungs. Data shown are log_2 normalized abundances of beta-hydroxybutyrate. Error bars show standard error of three replicates. Asterisks denote statistical significance as shown in key.

Figure S11. Metabolic pathway of arachidonic acid metabolism (mmu:00590). The entire metabolic pathway is shown to be increased in adults as compared to PND7 and PND14 mice. Proteins are indicated by rounded rectangles with protein names within, lipids and lipid metabolites are indicated by circles. Red font protein names indicates higher relative abundances were observed in adult samples, green font protein names indicates higher relative abundances were observed in PND7 samples, gray font protein names indicates that the species was not changing in a statistically significant manner. Bar graphs are provided for each protein with significance of differential expression across time indicated by stars. ($p<0.001$ - ***; $p<0.01$ - **; $p<0.05$ - *). The entire arachidonic acid metabolic pathway, starting with cleavage from intact phospholipids, is enriched in adults as compared to the younger samples. The underlying numerical data used to indicate protein expression patterns in the figure as well as statistical significance values is available in Tables S2.

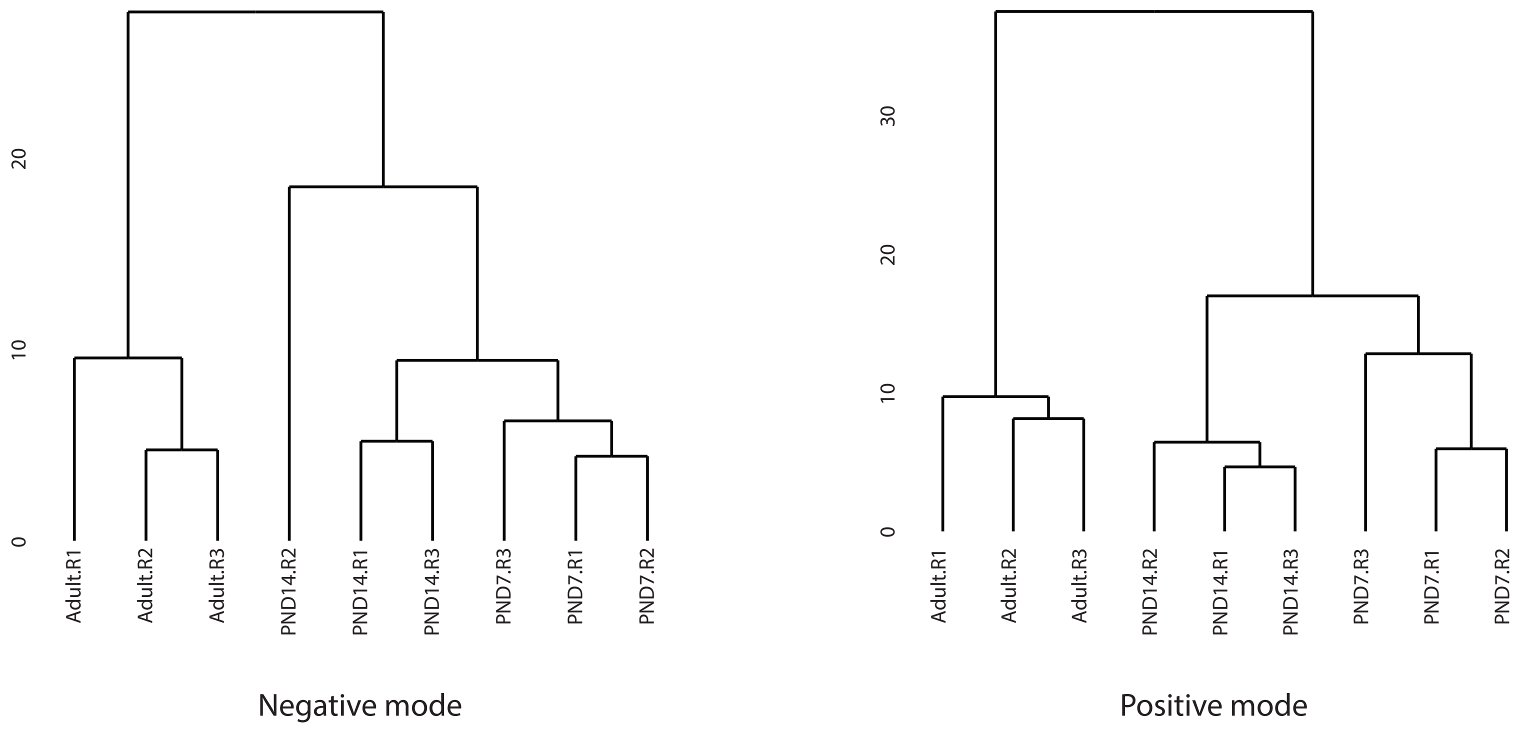

0
10
20
Adult.R1
Adult.R2
Adult.R3
PND14.R2
PND14.R1
PND14.R3
PND7.R3
PND7.R1
PND7.R2
Negative mode
0
10
20
30
Adult.R1
Adult.R2
Adult.R3
PND14.R2
PND14.R1
PND14.R3
PND7.R3
PND7.R1
PND7.R2
Positive mode

Figure S1

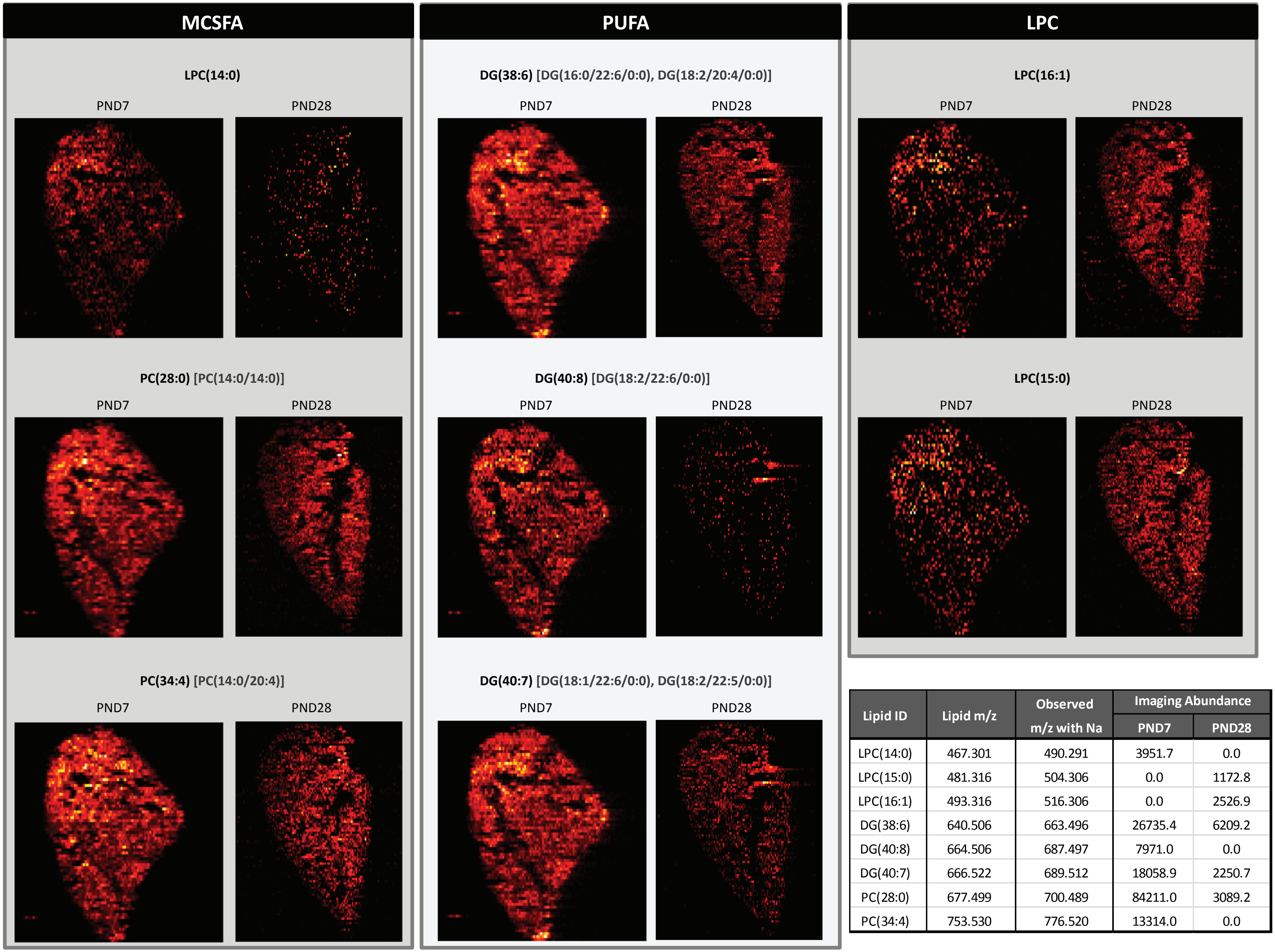

Lipid ID	Lipid m/z	Observed m/z with Na	Imaging Abundance	
			PND7	PND28
LPC(14:0)	467.301	490.291	3951.7	0.0
LPC(15:0)	481.316	504.306	0.0	1172.8
LPC(16:1)	493.316	516.306	0.0	2526.9
DG(38:6)	640.506	663.496	26735.4	6209.2
DG(40:8)	664.506	687.497	7971.0	0.0
DG(40:7)	666.522	689.512	18058.9	2250.7
PC(28:0)	677.499	700.489	84211.0	3089.2
PC(34:4)	753.530	776.520	13314.0	0.0

Figure S2

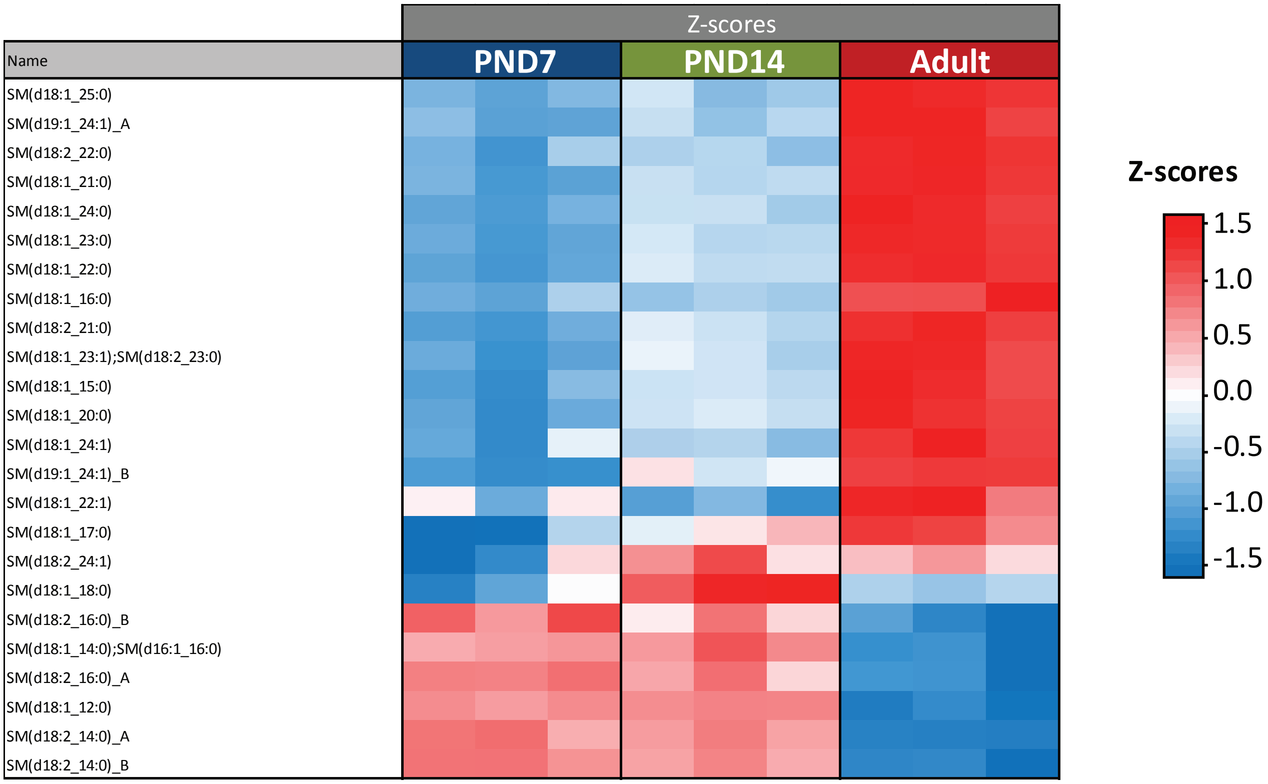
Z-scores
Name
PND7
PND14
Adult
SM(d18:1_25:0)
SM(d19:1_24:1)_A
SM(d18:2_22:0)
SM(d18:1_21:0)
SM(d18:1_24:0)
SM(d18:1_23:0)
SM(d18:1_22:0)
SM(d18:1_16:0)
SM(d18:2_21:0)
SM(d18:1_23:1);SM(d18:2_23:0)
SM(d18:1_15:0)
SM(d18:1_20:0)
SM(d18:1_24:1)
SM(d19:1_24:1)_B
SM(d18:1_22:1)
SM(d18:1_17:0)
SM(d18:2_24:1)
SM(d18:1_18:0)
SM(d18:2_16:0)_B
SM(d18:1_14:0);SM(d16:1_16:0)
SM(d18:2_16:0)_A
SM(d18:1_12:0)
SM(d18:2_14:0)_A
SM(d18:2_14:0)_B
Z-scores
1.5
1.0
0.5
0.0
-0.5
-1.0
-1.5

Figure S3

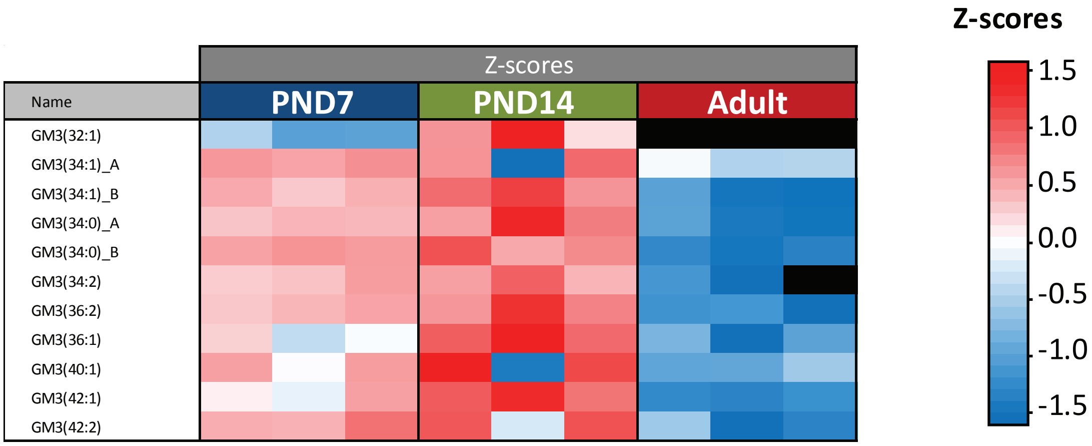
Z-scores
Z-scores
Name
PND7
PND14
Adult
GM3(32:1)
GM3(34:1)_A
GM3(34:1)_B
GM3(34:0)_A
GM3(34:0)_B
GM3(34:2)
GM3(36:2)
GM3(36:1)
GM3(40:1)
GM3(42:1)
GM3(42:2)
1.5
1.0
0.5
0.0
-0.5
-1.0
-1.5

Figure S4

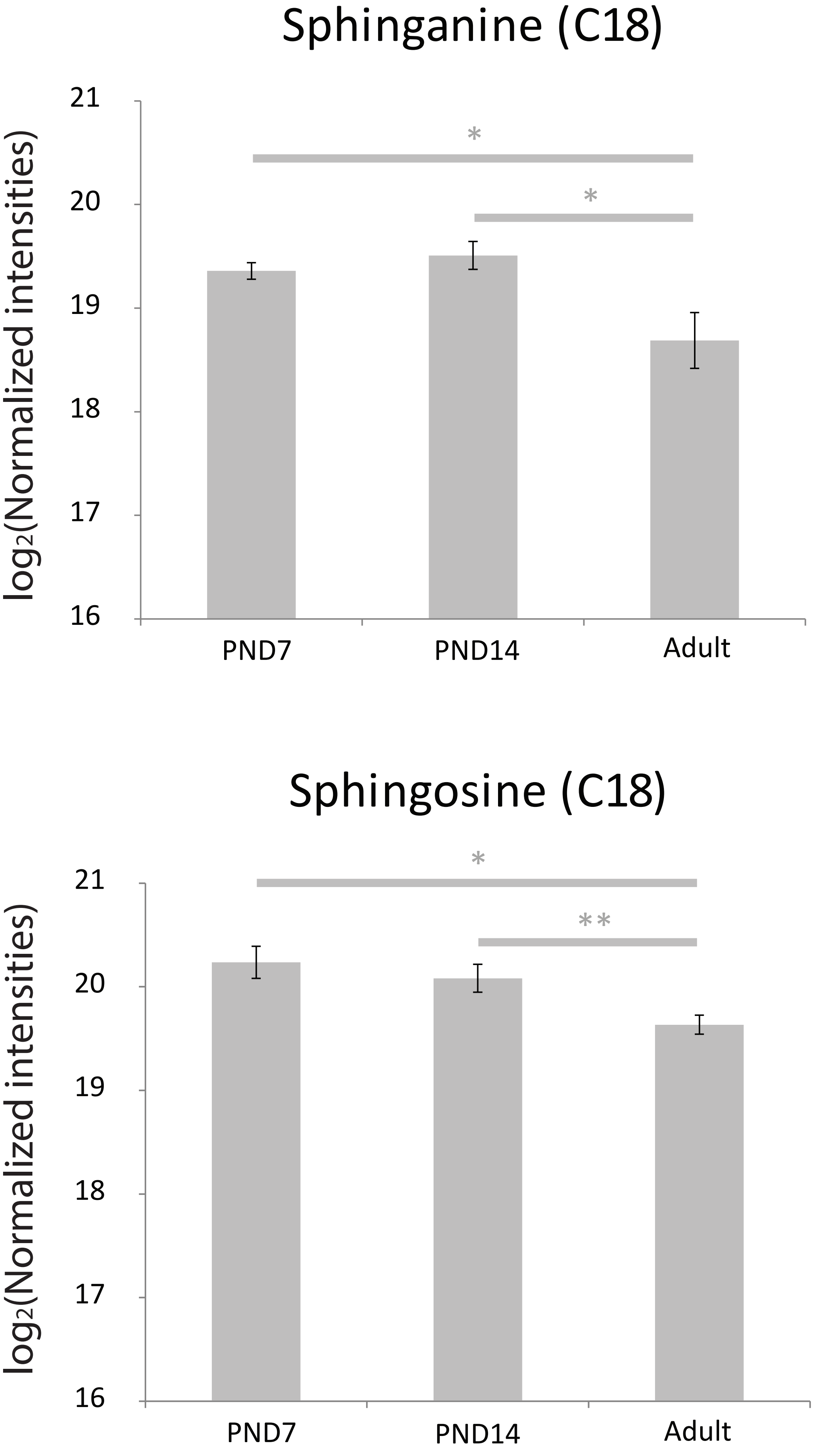

* pvalue<0.05
** pvalue<0.01
*** pvalue<0.001

Figure S5

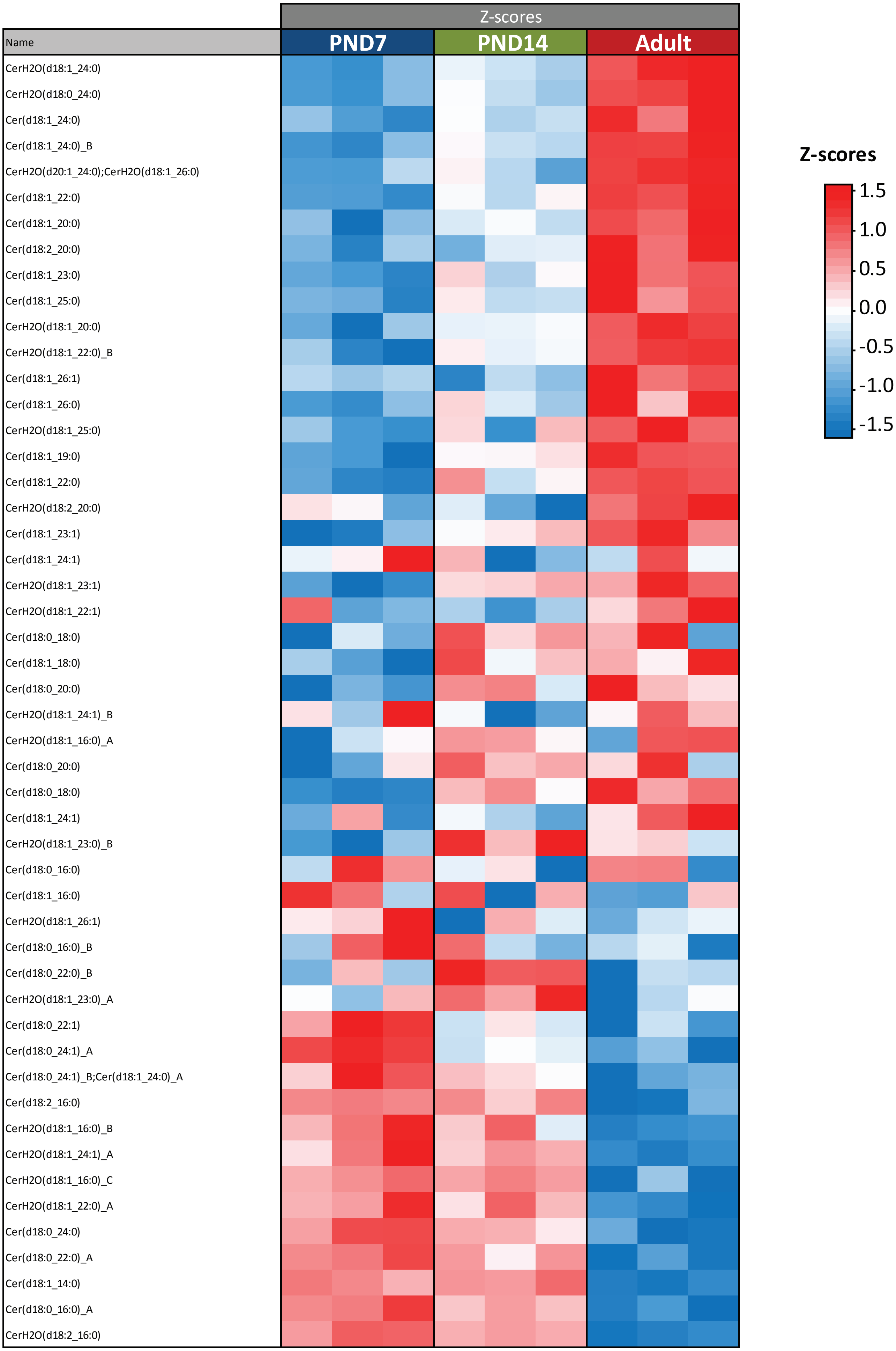
Z-scores
Name
PND7
PND14
Adult
CerH2O(d18:1_24:0)
CerH2O(d18:0_24:0)
Cer(d18:1_24:0)
Cer(d18:1_24:0)_B
CerH2O(d20:1_24:0);CerH2O(d18:1_26:0)
Cer(d18:1_22:0)
Cer(d18:1_20:0)
Cer(d18:2_20:0)
Cer(d18:1_23:0)
Cer(d18:1_25:0)
CerH2O(d18:1_20:0)
CerH2O(d18:1_22:0)_B
Cer(d18:1_26:1)
Cer(d18:1_26:0)
CerH2O(d18:1_25:0)
Cer(d18:1_19:0)
Cer(d18:1_22:0)
CerH2O(d18:2_20:0)
Cer(d18:1_23:1)
Cer(d18:1_24:1)
CerH2O(d18:1_23:1)
CerH2O(d18:1_22:1)
Cer(d18:0_18:0)
Cer(d18:1_18:0)
Cer(d18:0_20:0)
CerH2O(d18:1_24:1)_B
CerH2O(d18:1_16:0)_A
Cer(d18:0_20:0)
Cer(d18:0_18:0)
Cer(d18:1_24:1)
CerH2O(d18:1_23:0)_B
Cer(d18:0_16:0)
Cer(d18:1_16:0)
CerH2O(d18:1_26:1)
Cer(d18:0_16:0)_B
Cer(d18:0_22:0)_B
CerH2O(d18:1_23:0)_A
Cer(d18:0_22:1)
Cer(d18:0_24:1)_A
Cer(d18:0_24:1)_B;Cer(d18:1_24:0)_A
Cer(d18:2_16:0)
CerH2O(d18:1_16:0)_B
CerH2O(d18:1_24:1)_A
CerH2O(d18:1_16:0)_C
CerH2O(d18:1_22:0)_A
Cer(d18:0_24:0)
Cer(d18:0_22:0)_A
Cer(d18:1_14:0)
Cer(d18:0_16:0)_A
CerH2O(d18:2_16:0)
Z-scores
1.5
1.0
0.5
0.0
-0.5
-1.0
-1.5

Figure S6

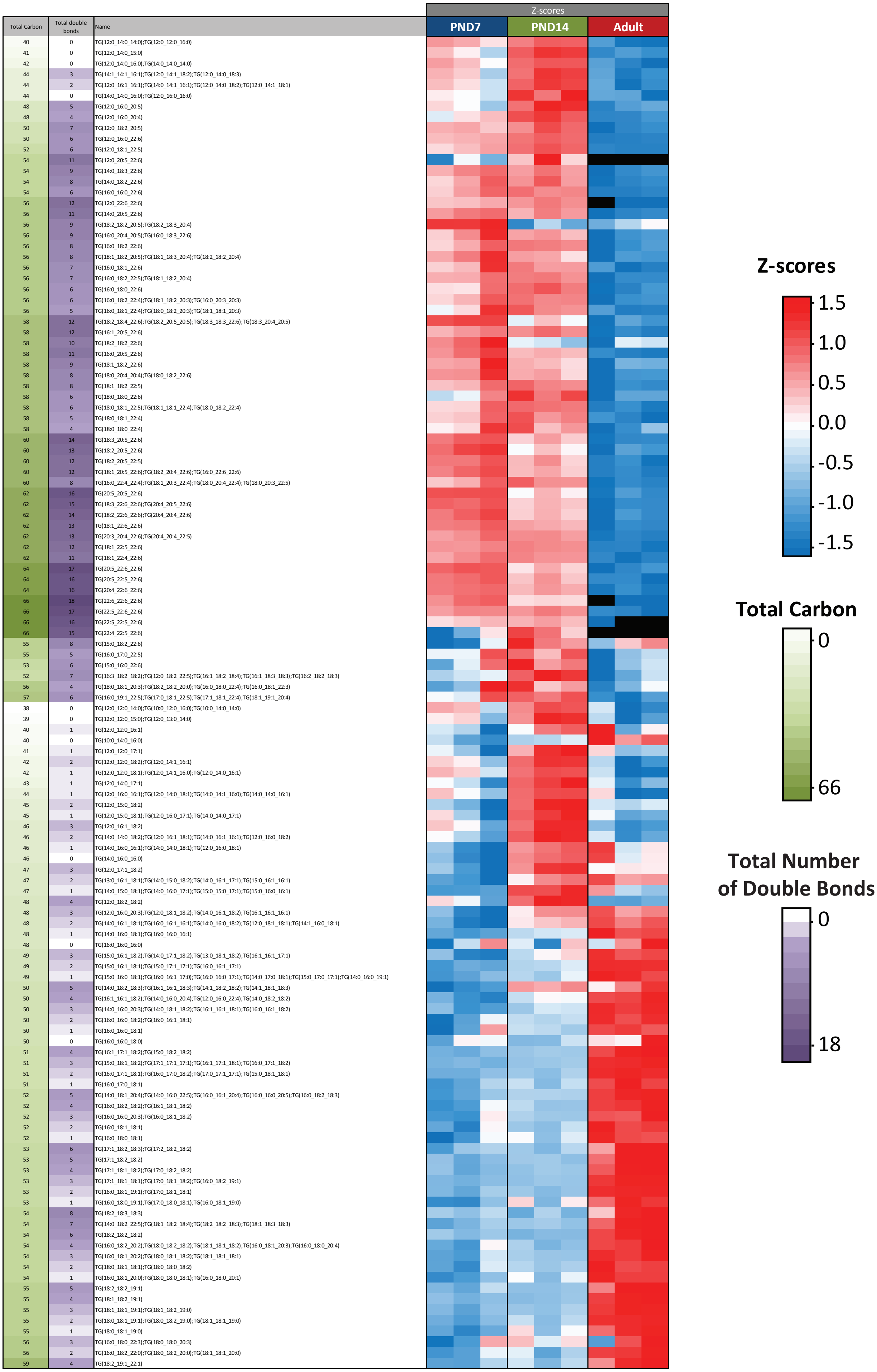
Z-scores
PND7
PND14
Adult
Total Carbon
Total double bonds
Name
40 0 TG(12:0_14:0_14:0);TG(12:0_12:0_16:0)
41 0 TG(12:0_14:0_15:0)
42 0 TG(12:0_14:0_16:0);TG(14:0_14:0_14:0)
44 3 TG(14:1_14:1_16:1);TG(12:0_14:1_18:2);TG(12:0_14:0_18:3)
44 2 TG(12:0_16:1_16:1);TG(14:0_14:1_16:1);TG(12:0_14:0_18:2);TG(12:0_14:1_18:1)
44 0 TG(14:0_14:0_16:0);TG(12:0_16:0_16:0)
48 5 TG(12:0_16:0_20:5)
48 4 TG(12:0_16:0_20:4)
50 7 TG(12:0_18:2_20:5)
50 6 TG(12:0_16:0_22:6)
52 6 TG(12:0_18:1_22:5)
54 11 TG(12:0_20:5_22:6)
54 9 TG(14:0_18:3_22:6)
54 8 TG(14:0_18:2_22:6)
54 6 TG(16:0_16:0_22:6)
56 12 TG(12:0_22:6_22:6)
56 11 TG(14:0_20:5_22:6)
56 9 TG(18:2_18:2_20:5);TG(18:2_18:3_20:4)
56 9 TG(16:0_20:4_20:5);TG(16:0_18:3_22:6)
56 8 TG(16:0_18:2_22:6)
56 8 TG(18:1_18:2_20:5);TG(18:1_18:3_20:4);TG(18:2_18:2_20:4)
56 7 TG(16:0_18:1_22:6)
56 7 TG(16:0_18:2_22:5);TG(18:1_18:2_20:4)
56 6 TG(16:0_18:0_22:6)
56 6 TG(16:0_18:2_22:4);TG(18:1_18:2_20:3);TG(16:0_20:3_20:3)
56 5 TG(16:0_18:1_22:4);TG(18:0_18:2_20:3);TG(18:1_18:1_20:3)
58 12 TG(18:2_18:4_22:6);TG(18:2_20:5_20:5);TG(18:3_18:3_22:6);TG(18:3_20:4_20:5)
58 12 TG(16:1_20:5_22:6)
58 10 TG(18:2_18:2_22:6)
58 11 TG(16:0_20:5_22:6)
58 9 TG(18:1_18:2_22:6)
58 8 TG(18:0_20:4_20:4);TG(18:0_18:2_22:6)
58 8 TG(18:1_18:2_22:5)
58 6 TG(18:0_18:0_22:6)
58 6 TG(18:0_18:1_22:5);TG(18:1_18:1_22:4);TG(18:0_18:2_22:4)
58 5 TG(18:0_18:1_22:4)
58 4 TG(18:0_18:0_22:4)
60 14 TG(18:3_20:5_22:6)
60 13 TG(18:2_20:5_22:6)
60 12 TG(18:2_20:5_22:5)
60 12 TG(18:1_20:5_22:6);TG(18:2_20:4_22:6);TG(16:0_22:6_22:6)
60 8 TG(16:0_22:4_22:4);TG(18:1_20:3_22:4);TG(18:0_20:4_22:4);TG(18:0_20:3_22:5)
62 16 TG(20:5_20:5_22:6)
62 15 TG(18:3_22:6_22:6);TG(20:4_20:5_22:6)
62 14 TG(18:2_22:6_22:6);TG(20:4_20:4_22:6)
62 13 TG(18:1_22:6_22:6)
62 13 TG(20:3_20:4_22:6);TG(20:4_20:4_22:5)
62 12 TG(18:1_22:5_22:6)
62 11 TG(18:1_22:4_22:6)
64 17 TG(20:5_22:6_22:6)
64 16 TG(20:5_22:5_22:6)
64 16 TG(20:4_22:6_22:6)
66 18 TG(22:6_22:6_22:6)
66 17 TG(22:5_22:6_22:6)
66 16 TG(22:5_22:5_22:6)
66 15 TG(22:4_22:5_22:6)
55 8 TG(15:0_18:2_22:6)
55 5 TG(16:0_17:0_22:5)
53 6 TG(15:0_16:0_22:6)
52 7 TG(16:3_18:2_18:2);TG(12:0_18:2_22:5);TG(16:1_18:2_18:4);TG(16:1_18:3_18:3);TG(16:2_18:2_18:3)
56 4 TG(18:0_18:1_20:3);TG(18:2_18:2_20:0);TG(16:0_18:0_22:4);TG(16:0_18:1_22:3)
57 6 TG(16:0_19:1_22:5);TG(17:0_18:1_22:5);TG(17:1_18:1_22:4);TG(18:1_19:1_20:4)
38 0 TG(12:0_12:0_14:0);TG(10:0_12:0_16:0);TG(10:0_14:0_14:0)
39 0 TG(12:0_12:0_15:0);TG(12:0_13:0_14:0)
40 1 TG(12:0_12:0_16:1)
40 0 TG(10:0_14:0_16:0)
41 1 TG(12:0_12:0_17:1)
42 2 TG(12:0_12:0_18:2);TG(12:0_14:1_16:1)
42 1 TG(12:0_12:0_18:1);TG(12:0_14:1_16:0);TG(12:0_14:0_16:1)
43 1 TG(12:0_14:0_17:1)
44 1 TG(12:0_16:0_16:1);TG(12:0_14:0_18:1);TG(14:0_14:1_16:0);TG(14:0_14:0_16:1)
45 2 TG(12:0_15:0_18:2)
45 1 TG(12:0_15:0_18:1);TG(12:0_16:0_17:1);TG(14:0_14:0_17:1)
46 3 TG(12:0_16:1_18:2)
46 2 TG(14:0_14:0_18:2);TG(12:0_16:1_18:1);TG(14:0_16:1_16:1);TG(12:0_16:0_18:2)
46 1 TG(14:0_16:0_16:1);TG(14:0_14:0_18:1);TG(12:0_16:0_18:1)
46 0 TG(14:0_16:0_16:0)
47 3 TG(12:0_17:1_18:2)
47 2 TG(13:0_16:1_18:1);TG(14:0_15:0_18:2);TG(14:0_16:1_17:1);TG(15:0_16:1_16:1)
47 1 TG(14:0_15:0_18:1);TG(14:0_16:0_17:1);TG(15:0_15:0_17:1);TG(15:0_16:0_16:1)
48 4 TG(12:0_18:2_18:2)
48 3 TG(12:0_16:0_20:3);TG(12:0_18:1_18:2);TG(14:0_16:1_18:2);TG(16:1_16:1_16:1)
48 2 TG(14:0_16:1_18:1);TG(16:0_16:1_16:1);TG(14:0_16:0_18:2);TG(12:0_18:1_18:1);TG(14:1_16:0_18:1)
48 1 TG(14:0_16:0_18:1);TG(16:0_16:0_16:1)
48 0 TG(16:0_16:0_16:0)
49 3 TG(15:0_16:1_18:2);TG(14:0_17:1_18:2);TG(13:0_18:1_18:2);TG(16:1_16:1_17:1)
49 2 TG(15:0_16:1_18:1);TG(15:0_17:1_17:1);TG(16:0_16:1_17:1)
49 1 TG(15:0_16:0_18:1);TG(16:0_16:1_17:0);TG(16:0_16:0_17:1);TG(14:0_17:0_18:1);TG(15:0_17:0_17:1);TG(14:0_16:0_19:1)
50 5 TG(14:0_18:2_18:3);TG(16:1_16:1_18:3);TG(14:1_18:2_18:2);TG(14:1_18:1_18:3)
50 4 TG(16:1_16:1_18:2);TG(14:0_16:0_20:4);TG(12:0_16:0_22:4);TG(14:0_18:2_18:2)
50 3 TG(14:0_16:0_20:3);TG(14:0_18:1_18:2);TG(16:1_16:1_18:1);TG(16:0_16:1_18:2)
50 2 TG(16:0_16:0_18:2);TG(16:0_16:1_18:1)
50 1 TG(16:0_16:0_18:1)
50 0 TG(16:0_16:0_18:0)
51 4 TG(16:1_17:1_18:2);TG(15:0_18:2_18:2)
51 3 TG(15:0_18:1_18:2);TG(17:1_17:1_17:1);TG(16:1_17:1_18:1);TG(16:0_17:1_18:2)
51 2 TG(16:0_17:1_18:1);TG(16:0_17:0_18:2);TG(17:0_17:1_17:1);TG(15:0_18:1_18:1)
51 1 TG(16:0_17:0_18:1)
52 5 TG(14:0_18:1_20:4);TG(14:0_16:0_22:5);TG(16:0_16:1_20:4);TG(16:0_16:0_20:5);TG(16:0_18:2_18:3)
52 4 TG(16:0_18:2_18:2);TG(16:1_18:1_18:2)
52 3 TG(16:0_16:0_20:3);TG(16:0_18:1_18:2)
52 2 TG(16:0_18:1_18:1)
52 1 TG(16:0_18:0_18:1)
53 6 TG(17:1_18:2_18:3);TG(17:2_18:2_18:2)
53 5 TG(17:1_18:2_18:2)
53 4 TG(17:1_18:1_18:2);TG(17:0_18:2_18:2)
53 3 TG(17:1_18:1_18:1);TG(17:0_18:1_18:2);TG(16:0_18:2_19:1)
53 2 TG(16:0_18:1_19:1);TG(17:0_18:1_18:1)
53 1 TG(16:0_18:0_19:1);TG(17:0_18:0_18:1);TG(16:0_18:1_19:0)
54 8 TG(18:2_18:3_18:3)
54 7 TG(14:0_18:2_22:5);TG(18:1_18:2_18:4);TG(18:2_18:2_18:3);TG(18:1_18:3_18:3)
54 6 TG(18:2_18:2_18:2)
54 4 TG(16:0_18:2_20:2);TG(18:0_18:2_18:2);TG(18:1_18:1_18:2);TG(16:0_18:1_20:3);TG(16:0_18:0_20:4)
54 3 TG(16:0_18:1_20:2);TG(18:0_18:1_18:2);TG(18:1_18:1_18:1)
54 2 TG(18:0_18:1_18:1);TG(18:0_18:0_18:2)
54 1 TG(16:0_18:1_20:0);TG(18:0_18:0_18:1);TG(16:0_18:0_20:1)
55 5 TG(18:2_18:2_19:1)
55 4 TG(18:1_18:2_19:1)
55 3 TG(18:1_18:1_19:1);TG(18:1_18:2_19:0)
55 2 TG(18:0_18:1_19:1);TG(18:0_18:2_19:0);TG(18:1_18:1_19:0)
55 1 TG(18:0_18:1_19:0)
56 3 TG(16:0_18:0_22:3);TG(18:0_18:0_20:3)
56 2 TG(16:0_18:2_22:0);TG(18:0_18:2_20:0);TG(18:1_18:1_20:0)
59 4 TG(18:2_19:1_22:1)
Z-scores
1.5
1.0
0.5
0.0
-0.5
-1.0
-1.5
Total Carbon
0
66
Total Number of Double Bonds
0
18

Figure S7

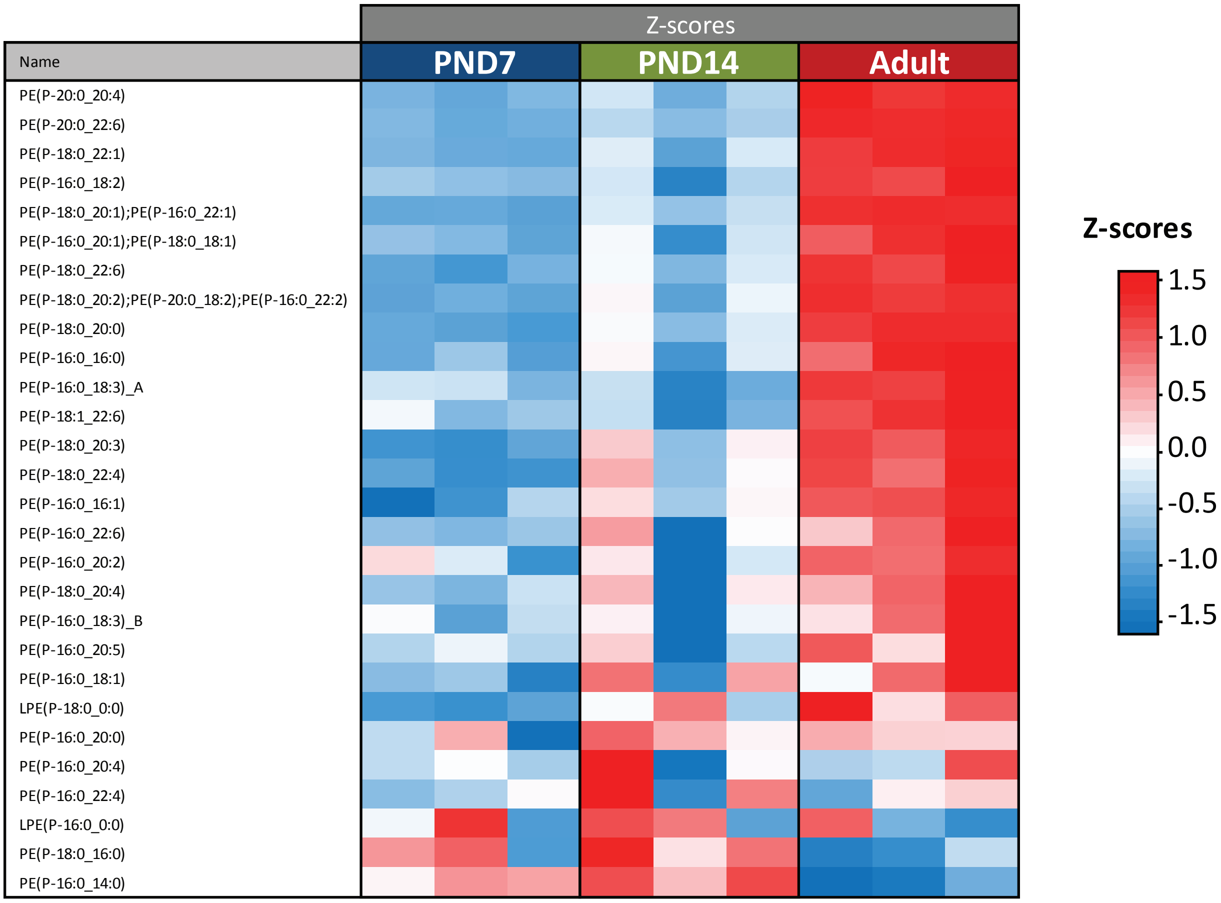
Z-scores
Name
PND7
PND14
Adult
PE(P-20:0_20:4)
PE(P-20:0_22:6)
PE(P-18:0_22:1)
PE(P-16:0_18:2)
PE(P-18:0_20:1);PE(P-16:0_22:1)
PE(P-16:0_20:1);PE(P-18:0_18:1)
PE(P-18:0_22:6)
PE(P-18:0_20:2);PE(P-20:0_18:2);PE(P-16:0_22:2)
PE(P-18:0_20:0)
PE(P-16:0_16:0)
PE(P-16:0_18:3)_A
PE(P-18:1_22:6)
PE(P-18:0_20:3)
PE(P-18:0_22:4)
PE(P-16:0_16:1)
PE(P-16:0_22:6)
PE(P-16:0_20:2)
PE(P-18:0_20:4)
PE(P-16:0_18:3)_B
PE(P-16:0_20:5)
PE(P-16:0_18:1)
LPE(P-18:0_0:0)
PE(P-16:0_20:0)
PE(P-16:0_20:4)
PE(P-16:0_22:4)
LPE(P-16:0_0:0)
PE(P-18:0_16:0)
PE(P-16:0_14:0)
Z-scores
1.5
1.0
0.5
0.0
-0.5
-1.0
-1.5

Figure S8

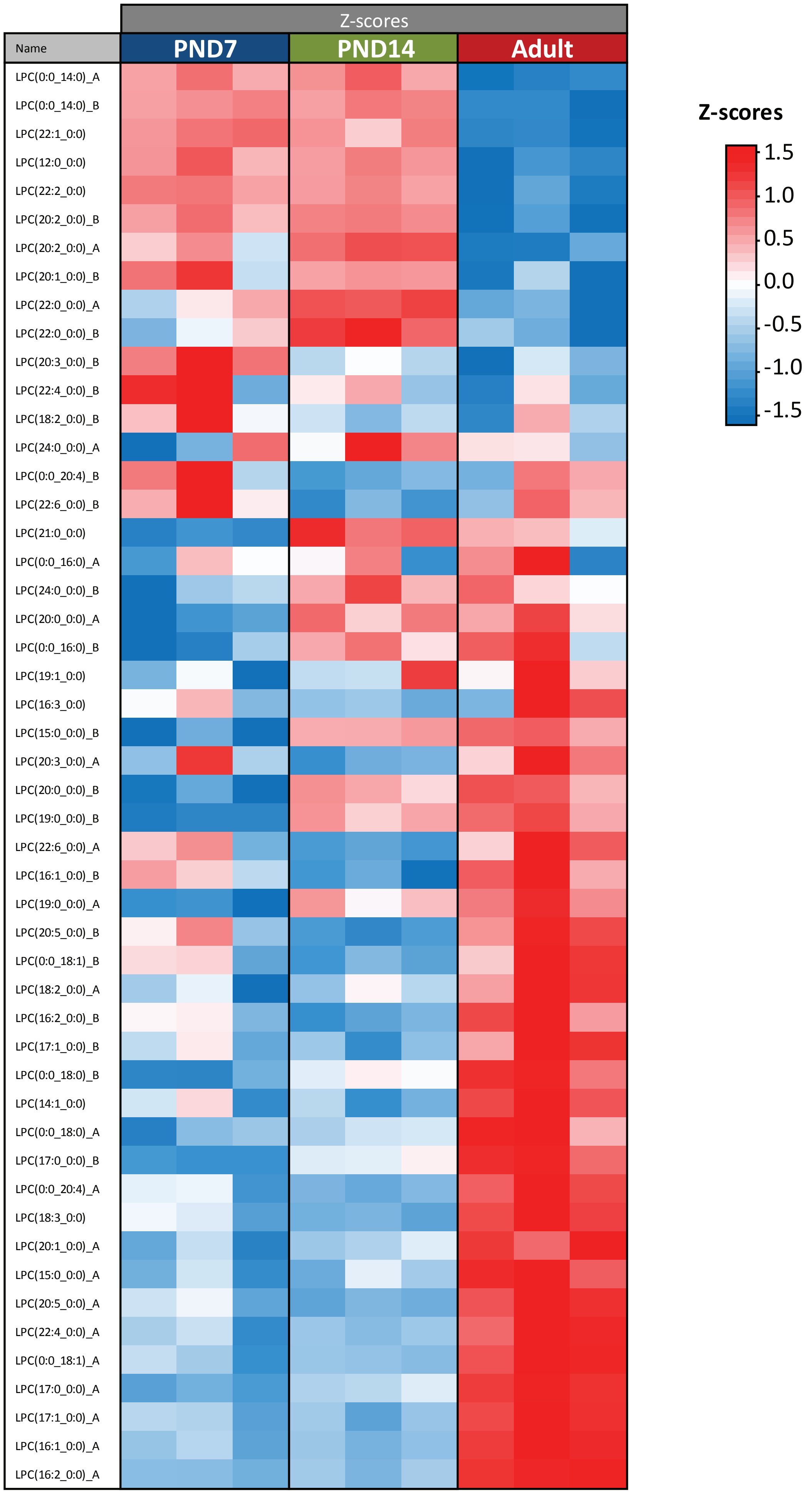
Z-scores
Name
PND7
PND14
Adult
LPC(0:0_14:0)_A
LPC(0:0_14:0)_B
LPC(22:1_0:0)
LPC(12:0_0:0)
LPC(22:2_0:0)
LPC(20:2_0:0)_B
LPC(20:2_0:0)_A
LPC(20:1_0:0)_B
LPC(22:0_0:0)_A
LPC(22:0_0:0)_B
LPC(20:3_0:0)_B
LPC(22:4_0:0)_B
LPC(18:2_0:0)_B
LPC(24:0_0:0)_A
LPC(0:0_20:4)_B
LPC(22:6_0:0)_B
LPC(21:0_0:0)
LPC(0:0_16:0)_A
LPC(24:0_0:0)_B
LPC(20:0_0:0)_A
LPC(0:0_16:0)_B
LPC(19:1_0:0)
LPC(16:3_0:0)
LPC(15:0_0:0)_B
LPC(20:3_0:0)_A
LPC(20:0_0:0)_B
LPC(19:0_0:0)_B
LPC(22:6_0:0)_A
LPC(16:1_0:0)_B
LPC(19:0_0:0)_A
LPC(20:5_0:0)_B
LPC(0:0_18:1)_B
LPC(18:2_0:0)_A
LPC(16:2_0:0)_B
LPC(17:1_0:0)_B
LPC(0:0_18:0)_B
LPC(14:1_0:0)
LPC(0:0_18:0)_A
LPC(17:0_0:0)_B
LPC(0:0_20:4)_A
LPC(18:3_0:0)
LPC(20:1_0:0)_A
LPC(15:0_0:0)_A
LPC(20:5_0:0)_A
LPC(22:4_0:0)_A
LPC(0:0_18:1)_A
LPC(17:0_0:0)_A
LPC(17:1_0:0)_A
LPC(16:1_0:0)_A
LPC(16:2_0:0)_A
Z-scores
1.5
1.0
0.5
0.0
-0.5
-1.0
-1.5

Figure S9

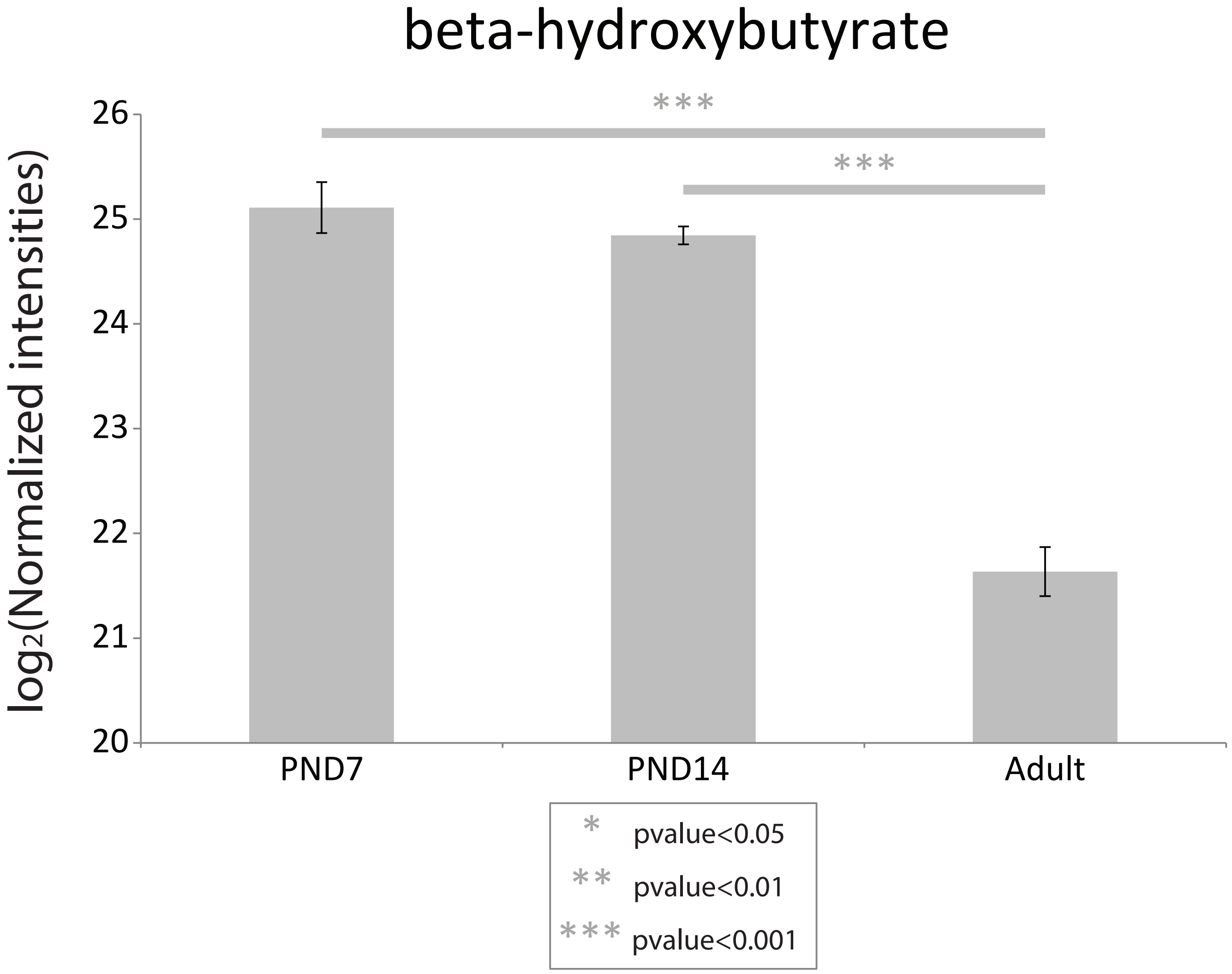
beta-hydroxybutyrate
***
***
26
25
24
23
22
21
20
log2(Normalized intensities)
PND7
PND14
Adult
* pvalue<0.05
** pvalue<0.01
*** pvalue<0.001

Figure S10

Arachidonic acid metabolism

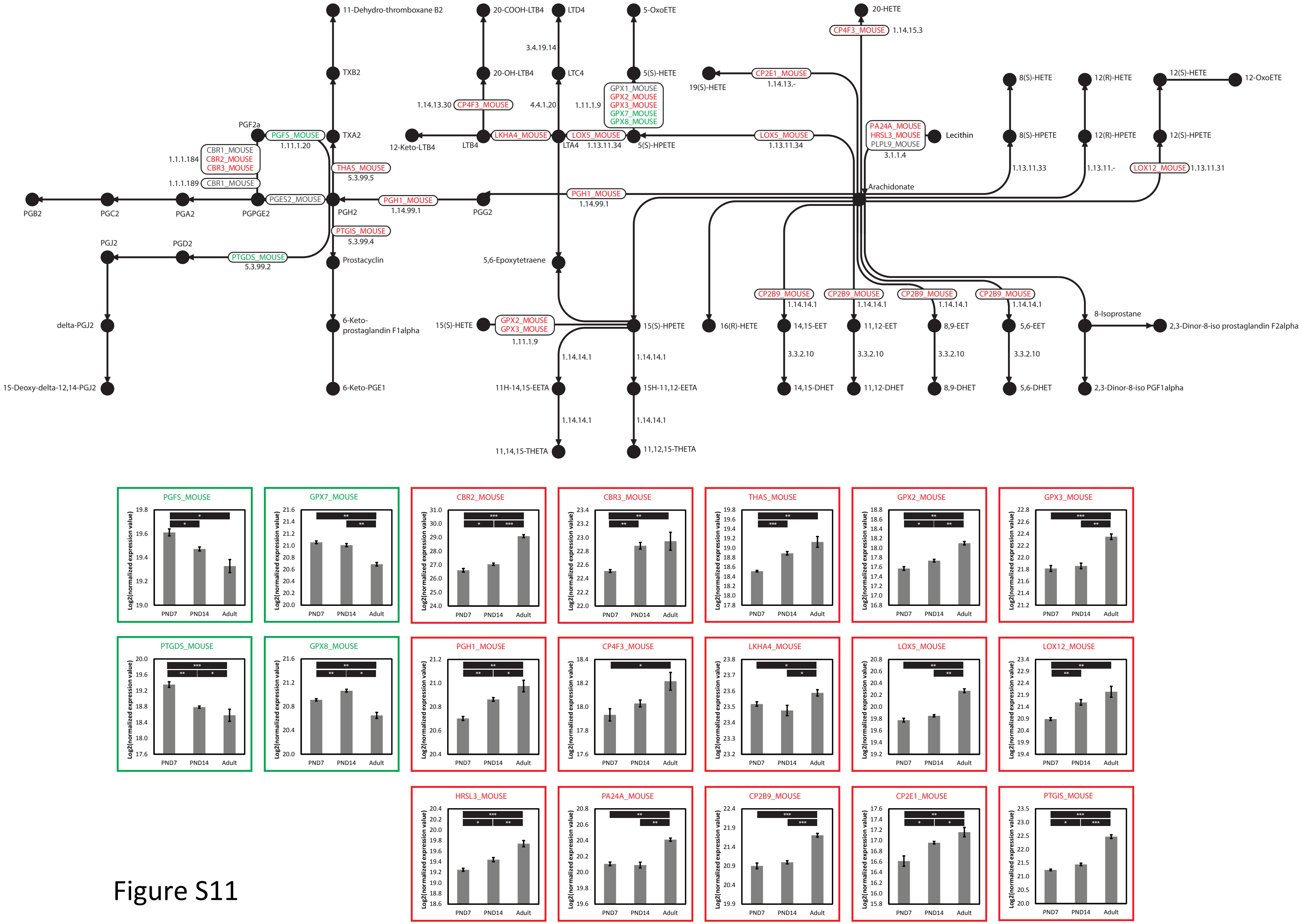

Figure S11
